# Circlator: automated circularization of genome assemblies using long sequencing reads

**DOI:** 10.1186/s13059-015-0849-0

**Published:** 2015-12-29

**Authors:** Martin Hunt, Nishadi De Silva, Thomas D. Otto, Julian Parkhill, Jacqueline A. Keane, Simon R. Harris

**Affiliations:** Wellcome Trust Sanger Institute, Wellcome Trust Genome Campus, Cambridge, CB10 1SA UK

**Keywords:** Assembly, Circularization

## Abstract

The assembly of DNA sequence data is undergoing a renaissance thanks to emerging technologies capable of producing reads tens of kilobases long. Assembling complete bacterial and small eukaryotic genomes is now possible, but the final step of circularizing sequences remains unsolved. Here we present Circlator, the first tool to automate assembly circularization and produce accurate linear representations of circular sequences. Using Pacific Biosciences and Oxford Nanopore data, Circlator correctly circularized 26 of 27 circularizable sequences, comprising 11 chromosomes and 12 plasmids from bacteria, the apicoplast and mitochondrion of *Plasmodium falciparum* and a human mitochondrion. Circlator is available at http://sanger-pathogens.github.io/circlator/.

**Electronic supplementary material:**

The online version of this article (doi:10.1186/s13059-015-0849-0) contains supplementary material, which is available to authorized users.

## Background

The challenge of de novo sequence assembly has existed ever since the invention of the first automated DNA sequencers. The assembly of early genome sequence data was largely based on two strategies: BAC/YAC tiling or whole-genome shotgun [1]. Although these strategies allow production of high-quality sequences, which are often used as reference genomes today, they are both slow and expensive, and the final stage of completing and, where necessary, circularizing the sequences requires laborious and costly manual finishing. The arrival of high-yielding, short-read sequencing technologies drastically reduced the time and cost required to generate high-depth whole-genome sequencing data ideal for identification of population variation. However, de novo genome assemblies from these data are typically too fragmented for genome completion to be practical, and consequently most short-read assembly algorithms do not tackle issues such as circularization of completed genomes.

The recent availability of high-throughput, long-read (up to tens of kilobases) sequencing technologies, in particular from Pacific Biosciences (PacBio) and Oxford Nanopore Technologies (ONT), has again improved the contiguity of automated de novo assemblies to the point where production of a single contig per DNA molecule is now possible on an unprecedented scale for bacterial and small eukaryotic genomes [2]. In comparison with short-read technologies, the per base error rate of PacBio and particularly ONT reads is high, but this has been mitigated by taking advantage of the high yield and random error model of these sequencers. By correcting errors in the raw sequencing reads using self-mapping, high-quality long sequences can be produced that are then contiguated using an overlap layout consensus assembly approach (for example, using HGAP [3], PBcR [4], and SPRAI [5]).

Although these new technologies raise the prospect of routine automated completion of genome sequences, current long-read assembly software still typically assumes that the contigs they produce are linear. In contrast, the genome of almost every species contains at least one circular DNA structure, such as bacterial chromosomes and plasmids and the plastid and mitochondrial genomes of eukaryotes. Correct completion and circularization of these molecules is essential if they are to be used routinely in clinical practice. Whole-genome sequencing is already providing improved resolution in bacterial epidemiology [6] and allowing in silico prediction of antimicrobial resistance [7]. Many important antimicrobial resistance and virulence determinants are carried on plasmids, illustrating the importance of having complete and accurate information for these circular sequences. Similarly, in humans, the mitochondrial genome has been implicated in controlling phenotypes such as depression [8, 9], Leber hereditary optic neuropathy [10], and myopathy and diabetes mellitus [11].

**Fig. 1:**
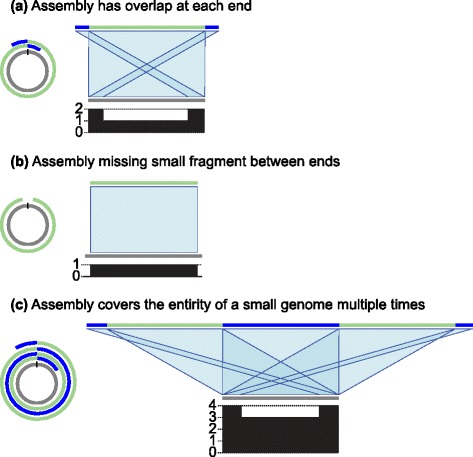
Typical issues in contigs produced by long-read assemblers representing circular sequences. In each example, the assembly is in a single contig, colored with a mix of *green* and *blue*, and the reference is shown in *gray*. Matches between the reference and assembly are shown in *light blue*. The *plot* below each reference sequence shows the number of matches to the assembly at each position of the reference sequence. **a** The contig has low-quality ends representing the same sequence, which needs resolving into one sequence. **b** The contig has missing sequence. **c** A small circular sequence is assembled into multiple tandem copies

It is, therefore, clearly important to be able to automatically produce accurate representations of circular DNA structures. However, the linear contigs produced by assembly programs to represent circular DNA structures can contain errors. Near-identical overlaps are often found at each end of the contig, which require significant manual intervention to resolve (Fig. 1a). Alternatively, the sequence may be incomplete, with a short sequence that would join the contig ends absent (Fig. 1b). Finally, when the sequence being assembled is shorter than the length of some reads, it may contain significant misassemblies in the form of multiple duplications of the entire circular molecule (Fig. 1c). Currently, there are two main approaches to resolving the circular structure, based on using BLAST [12] and Minimus2 [13] to identify the common sequence at each contig end, which we shall refer to as simply BLAST and Minimus2 for the remainder of the manuscript. In our evaluations, these methods fail to address the significant incorrect structural representations produced by de novo assemblers and therefore require significant further manual examination to produce correct representations of circular DNA structures.

In this paper, we introduce Circlator, a post-assembly improvement toolkit for producing correctly represented circular DNA structures. It uses local assemblies of corrected long reads at contig ends to circularize contigs. This avoids searching for a sequence in common between low-quality contig ends, and allows circularization even when overlaps are not present. We evaluated Circlator using examples from a wide range of bacteria, a human genome, and a *Plasmodium* sample and show that it outperforms current methods.

## Results and discussion

The Circlator workflow (Fig. 1, Additional file 1: Figure S1) consists of iteratively merging together contigs followed by running local assemblies of corrected reads that align to contig ends. These local assemblies are used to identify circular sequences, each of which is transformed into a linear representation of a circular sequence. Next, the assembly is cleaned by removing small contigs and non-circular contigs that are completely contained within the sequence of another contig. Finally, each circular contig has its start position set to a specified gene if present (by default *dnaA*), otherwise the start of a predicted gene near its center is used.

To test the applicability and scalability of Circlator, it was evaluated on 14 bacterial genomes, the circular apicoplast and mitochondrion genomes of the malaria parasite *Plasmodium falciparum*, and the mitochondrion genome of *Homo sapiens*. Finally, we evaluated Circlator on an assembly of the bacterium *Escherichia coli* based on Oxford Nanopore data. In all cases, Circlator was compared against the BLAST and Minimus2 circularization methods described in the ‘Methods’ section. Comparisons of all reference sequences and input and output assemblies are shown in Additional file 1: Figures S2–19. Default settings were used for all programs, except where noted for the nanopore and *P. falciparum* data.

### Bacterial PacBio data

The evaluated panel of 14 bacterial strains included both Gram-positive and -negative species selected from the National Collection of Type Cultures (NCTC) 3000 project [14] on the basis of there being high-quality reference genome sequences of the same strains available for comparison (see Additional file 2: Table S1 for sample and reference genome accession numbers). In total, the reference genomes of the sequenced strains comprised 14 chromosomes and 14 plasmids. A per strain summary of the merging and circularization of the three evaluated methods is shown in Table 1, and a more detailed version is given in Additional file 2: Table S2.

**Table 1:** Summary of results on 14 bacterial genome assemblies

Species	Ref	HGAP	Circularizable contigs^a,b^			Correctly circularized^a^			Errors^c^		
NCTC ID	contigs	contigs	BLAST	Circlator	Minimus2	BLAST	Circlator	Minimus2	BLAST	Circlator	Minimus2
*Bacillus subtilis*	1	19	1	1	1	1	1	1	5	1	2
NCTC3610			(0,1)	(0,1)	(0,1)	(0,1)	(0,1)	(0,1)			
*Clostridium difficile*	2	11	0	0	0	0	0	0	0	0	0
NCTC13307			(0,0)	(0,0)	(0,0)	(0,0)	(0,0)	(0,0)			
*Staphylococcus epidermidis*	7	9	8	8	8	7	8	4	0	0	4
NCTC13360			(1,7)	(1,7)	(1,7)	(1,6)	(1,7)	(1,3)			
*Enterobacter cloacae*	3	8	1	2	2	1	1	1	0	0	0
NCTC10005			(0,1)	(1,1)	(1,1)	(0,1)	(0,1)	(0,1)			
*Salmonella* Typhimurium	2	7	1	1	2	1	1	1	2	0	1
NCTC13348			(0,1)	(0,1)	(1,1)	(0,1)	(0,1)	(0,1)			
*Yersinia enterocolitica*	2	4	1	1	1	1	1	1	0	0	0
NCTC10963			(1,0)	(1,0)	(1,0)	(1,0)	(1,0)	(1,0)			
*Staphylococcus aureus*	1	3	2	2	2	2	2	1	0	0	0
NCTC10833			(1,1)	(1,1)	(1,1)	(1,1)	(1,1)	(0,1)			
*Staphylococcus aureus*	3	2	1	1	1	0	1	0	0	0	0
NCTC13626			(1,0)	(1,0)	(1,0)	(0,0)	(1,0)	(0,0)			
*Salmonella* Enteritidis	2	2	2	2	2	2	2	1	0	0	0
NCTC13349			(1,1)	(1,1)	(1,1)	(1,1)	(1,1)	(0,1)			
*Legionella pneumophila*	1	2	1	1	1	0	1	0	0	0	0
NCTC11192			(1,0)	(1,0)	(1,0)	(0,0)	(1,0)	(0,0)			
*Staphylococcus aureus*	1	1	1	1	1	1	1	0	0	0	0
NCTC13616			(1,0)	(1,0)	(1,0)	(1,0)	(1,0)	(0,0)			
*Staphylococcus aureus*	1	1	1	1	1	1	1	0	0	0	0
NCTC13277			(1,0)	(1,0)	(1,0)	(1,0)	(1,0)	(0,0)			
*Bordetella pertussis*	1	1	1	1	1	1	1	0	0	0	0
NCTC13251			(1,0)	(1,0)	(1,0)	(1,0)	(1,0)	(0,0)			
*Salmonella bongori*	1	1	1	1	1	0	1	0	0	0	0
NCTC12419			(1,0)	(1,0)	(1,0)	(0,0)	(1,0)	(0,0)			
Total	28	71	22	23	24	18	22	10	7	1	7

^a^The first and second numbers in parentheses are counts of contigs corresponding to chromosomes and plasmids, respectively

^b^A contig is defined as circularizable if it includes the entire sequence of a chromosome or plasmid, irrespective of the presence or size of an overlap between its start and end

^c^All errors except for those on sample NCTC13360 were false circularizations, where a tool attempted to circularize a contig that should not be circular. All four errors made on sample NCTC13360 were incorrect circularizations, where an attempt was made to circularize a circular contig, but the output contained errors

#### Assembly

Assembly of our panel of bacterial genomes using HGAP produced a total of 71 contigs, of which 10 represented complete chromosome sequences and a further 12 represented complete plasmid sequences. Four of these plasmids were not present in the corresponding reference genomes and one plasmid from the reference genome of NCTC13626 was not represented in the HGAP assembly of the same strain. Comparison of the HGAP assemblies to the reference genomes showed that in four cases large regions of the assembled chromosomes were inverted relative to the reference genome. We suspect these represent misassemblies, but assessed that these inversions did not affect the possibility of circularizing the assembled chromosomes.

#### Merging

After merging, Circlator reduced the total number of contigs in the assemblies to 52, while Minimus2 made more merges, reducing the number of contigs to 48. In all cases, manual inspection confirmed the merges to be correct.

#### Circularizable contigs

Contigs were considered circularizable if they included the entire sequence of a chromosome or plasmid, irrespective of the presence or size of an overlap between the two ends of the contig. Under this definition, 22 contigs (10 chromosomes and 12 plasmids) were circularizable in the initial HGAP assemblies, and as a result of merging contigs, Circlator produced one and Minimus2 two extra circularizable chromosomes. All other chromosomes and plasmids were in multiple contigs, so could not be circularized. Therefore, the numbers of circularizable contigs for the three methods were 23 (11 chromosomes and 12 plasmids) for Circlator, 24 (12 chromosomes and 12 plasmids) for Minimus2 and 22 (10 chromosomes and 12 plasmids) for BLAST.

#### Circularization

Circlator correctly circularized 22 of 23 circularizable contigs. In comparison, Minimus2 correctly circularized 10/24, and BLAST 18/22. Minimus2 performed particularly badly at circularizing chromosomal contigs, for which it succeeded in only 2 of 12 cases.

Where assemblies are fragmented, the possibility of erroneously circularizing non-circular contigs arises, hereafter termed false circularization. False circularization is of particular concern because one cause of assembly fragmentation is the presence of large repeat sequences in the assembled DNA. Contig breaks are often associated with these repeats, and therefore the presence of overlapping sequence at both ends of a contig does not necessitate that the contig is circular. Such a situation is particularly problematic for overlap approaches to circularization. Indeed, the majority of false circularizations made by the methods in our comparison occurred in one sample, the *Bacillus subtilis* strain NCTC3610, whose assembly was the most fragmented of our test panel. For this sample, Minimus2 falsely circularized two fragments of the chromosome, and BLAST falsely circularized five contigs including four small contigs representing repeat sequences and one fragment of the chromosome. The only false circularization made by Circlator was also in NCTC3610, where it circularized a small contig representing a repeat sequence. In total, Minimus2 falsely circularized three contigs, and BLAST falsely circularized seven.

Assembly of long reads without accounting for circularization can provide particularly problematic results for small plasmids whose length is shorter than the length of the reads used to assemble it. This can lead to the production of contigs containing the entire sequence of the plasmid two or more times (Fig. 1c). Circlator and our iterative BLAST approach correctly identified these occurrences and collapsed the plasmid sequences down to a single copy, whereas Minimus2 recognized only the first copy of the repeat sequence at each end of the contig, leading to the creation of an incorrectly circularized contig in some cases (for example, the plasmids of sample NCTC13360, as shown in Additional file 1: Figure S13).

#### Polishing

After circularization with any of the three methods implemented here, it is important to correct errors in the assembly using raw sequencing reads, since it can contain single base errors and small insertions and deletions. For PacBio data, this polishing step is usually carried out using the Quiver [3] algorithm included in the SMRT-Analysis software package. Nanopolish [15] can be used to correct errors using nanopore data.

Quiver was run on the output of HGAP, and on the output of each of the circularization programs. All assemblies, both pre- and post-Quiver, were evaluated using QUAST [16] version 2.3 with the options --gage -R to use a reference sequence and run the GAGE [17] analysis. Complete QUAST results are given in Additional file 2: Table S3. Summary plots are shown in Additional file 1: Figure S20, where it can be seen that, after running Quiver, Circlator generally produces higher-quality assemblies than the other approaches. These plots also highlight that assembly polishing using Quiver is critical, regardless of which circularization method is used.

### *P. falciparum* apicoplast and mitochondrion

Another test set is the *P. falciparum* genome that consists of 14 linear chromosomes plus circular mitochondrion and apicoplast sequences. We evaluated the circularization tools using data from the reference strain 3D7 [18]. Since the latest available version of the apicoplast has a large deletion caused by a near-identical inverted repeat, we generated an improved apicoplast sequence to use as a reference sequence when determining the accuracy of the circularization tools (for details see Additional file 1).

**Fig. 2:**
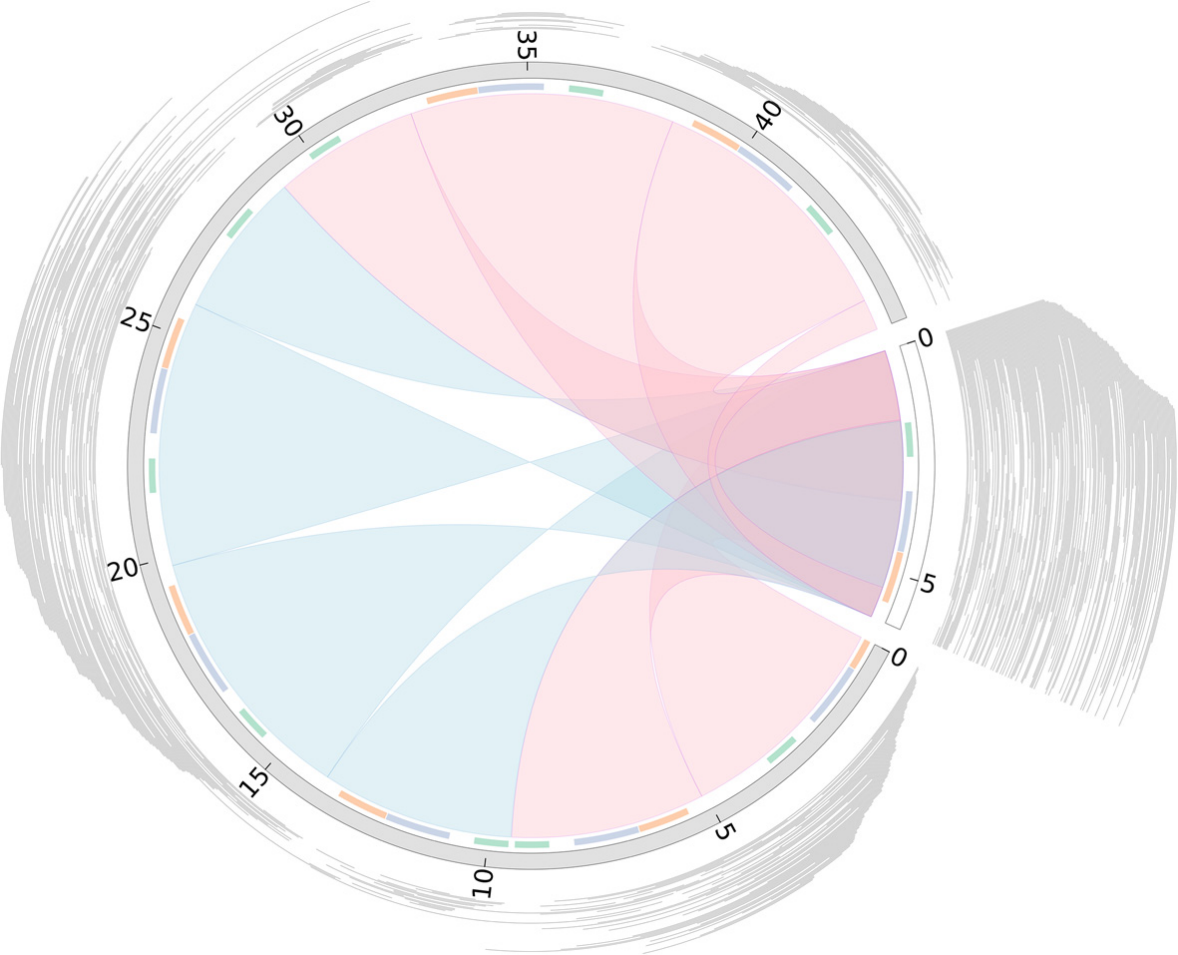
Comparison of HGAP assembly of *P. falciparum* apicoplast and Circlator output. The HGAP and Circlator assemblies are shown in gray and white, respectively, with the numbers showing the lengths in kilobases. Nucmer matches between the genomes are shown as blue (hits in the same orientation) and pink (hits in opposing orientations). Matches to the three apicoplast genes, *cox1* (blue), *cox3* (green), and *cob* (orange), are shown as a colored track inside the assemblies. The corrected reads mapped to each of the assemblies are shown in gray outside the assemblies. This figure was generated using Circos [31]

Reads from 11 PacBio SMRT cells (accessions ERR951787 to ERR951797 inclusive) were assembled using HGAP. Contigs matching the existing apicoplast and mitochondrion reference sequences were identified by taking all hits reported by nucmer (with default settings for nucmer, and delta-filter -i 95 -l 1000). The resulting two contigs, one for each of the apicoplast and mitochondrion, were input to the Minimus2 and BLAST methods. Circlator was run using the same two contigs, together with all corrected reads that mapped to those contigs using BWA MEM [19] with the option -x pacbio. Since the reads were low coverage, the kmer used by SPAdes [20] was set to 101 (with Circlator option --assemble_spades_k 101) for it to output complete assemblies. All three tools correctly circularized the apicoplast. Figure 2 shows a comparison of the HGAP assembly, which comprises many copies of the apicoplast sequence, against the output of Circlator. Minimus2 and BLAST attempted to circularize the mitochondrion, but failed to recognize the multiple copies in the input contig, and output several copies of the sequence. Of the three tools, only Circlator correctly circularized the mitochondrion sequence (Additional file 1: Figure S21).

### *H. sapiens* mitochondrion

To determine the applicability of circularization to the human mitochondrial genome, a test assembly, and set of corrected PacBio reads was generated from a shotgun sequence dataset (accession numbers SRR1304331 to SRR1304530 inclusive). To extract just the reads that correspond to the mitochondrion genome, first the reads were mapped to the reference GRCh38 mitochondrion sequence (NC_012920.1) using BWA MEM with the option -x pacbio. To remove false positives, the reads that mapped were then mapped to the entire GRCh38 genome using BWA MEM with the same settings. Only reads with a primary match to the mitochondrion were retained, and assembled with PBcR version 8.3rc2 using the options -maxCoverage 1000 -length 500 -partitions 200 genomeSize=16569. PBcR output a single contig, containing more than two copies of the mitochondrion sequence. Minimus2 attempted to circularize the contig but produced errors, whereas BLAST and Circlator correctly circularized this contig.

### *E. coli* nanopore data

We explored the possibility of circularizing a nanopore assembly, which is more challenging than PacBio because of the higher error rate in the reads and assembly contigs, using *Escherichia coli* ONT MinION data [15]. We used the two-dimensional reads available for download from the PBcR wiki page, which were already converted to FASTQ format using poretools [21]. We ran a de novo assembly of the reads using PBcR, to generate the contigs and corrected reads required as input to Circlator, using the data and instructions at [22] and checkout revision 4642 of the source code (see Additional file 1 for more details). Since the assembly and corrected reads are of lower quality than those of PacBio, it was required to make Circlator more permissive when matching the input assembly contigs to the SPAdes contigs generated by Circlator using the options --merge_min_id 85 --merge_breaklen 1000. These change the default MUMmer [23] parameters of Circlator, which are tuned to PacBio data, by passing -b 1000 to nucmer and -i 85 to delta-filter instead of the default -b 500 and -i 95. This lowers the minimum percentage identity from 95 to 85 (-i 85) and helps to extend hits through poorly aligned regions (-b 1000). Minimus2 and BLAST failed to circularize the assembly; however, Circlator successfully produced a correctly circularized genome sequence.

### Evaluation of user-defined parameters

In addition to using the default options of Circlator, we investigated the effects of changing eight key parameters in isolation on each of the 14 NCTC datasets, and on the Minion data, totaling 1848 runs of Circlator. The option that had the greatest effect on the results was --b2r_length_cutoff, which determines the cutoff between short and long contigs and consequently the length of contig ends that are reassembled (as described below in the ‘Read filtering and local assembly’ section of ‘Methods’). The other seven parameters that were varied all related to the contig merging and subsequent circularization stages of Circlator. The number of contig merges and circularized contigs changed only when extreme values were chosen, and in most cases, the results remained unchanged. For more details, see the Supplementary text, Additional file 2: Tables S4 and S5, Additional file 1: Table S6, and Additional file 1: Figures S22–S32. To aid troubleshooting when choosing parameters, Circlator outputs files compatible with ACT [24] that allow the user to compare the input assembly with the SPAdes reassembled contigs.

## Conclusions

Here we have presented the first tool that automatically resolves circular genome assemblies. Since circularization was the only remaining stage of genome assembly that required manual work, Circlator completes the automation of the process of assembling raw reads into a finished genome sequence. It was successfully applied to a wide range of species and different technologies and outperformed existing semi-automatic methods. In conclusion, Circlator provides the final step in the automated production of reference-quality long-read genome assemblies.

## Methods

### Circlator algorithm

The details of the Circlator algorithm and implementation are as follows.

#### Read filtering and local assembly

Circlator takes as input an assembly in FASTA format and the corrected reads that were used to produce that assembly in FASTA or FASTQ format. Both files are produced by common long-read assemblers, such as HGAP, PBcR, and SPRAI. The corrected reads are mapped to the assembly with BWA MEM using the -x pacbio option. Reads are then filtered for use in subsequent steps as follows (Additional file 1: Figure S33). For long contigs (by default, of length at least 100,000 bp), only reads that map to the first and last 50,000 bases of the contig are retained. A read that maps over the position 50,000 bases from either end of the contig is trimmed, so that only the part of the read mapping within 50,000 bases from the end of the contig is retained, provided the read is at least 250 bases long after trimming. The remaining reads, which are either mapped to short contigs (<100,000 bp) or are unmapped, are all retained. These filtered reads are then assembled using SPAdes with the options --careful --only-assembler to disable SPAdes’ own correction algorithm and assemble with high stringency. The longest allowed kmer length of 127 is used to maximize the contiguity of the assembly; however, SPAdes can fail with this kmer length if the read coverage is low. If SPAdes fails then the kmer length is reduced until an assembly is produced, using values 121, 111, 101, 95, 91, 85, 81, 75, and 71.

#### Contig merging

**Fig. 3:**
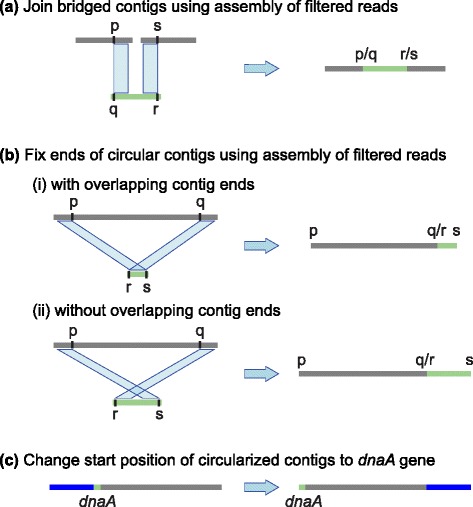
Key stages of the Circlator pipeline. **a** Before circularization, input contigs are merged using de novo assemblies of filtered reads. **b** Circular contigs are resolved using matches to contigs assembled from filtered reads. **c** Circularized contigs are rearranged to start at the *dnaA* gene, or a different gene specified by the user

The contigs of the resulting SPAdes assembly are aligned to the original assembly using the nucmer program of MUMmer with options --maxmatch --diagdiff 25, and hits with identity of at least 95 % are retained using delta-filter -i 95. The alignments to each SPAdes contig are analyzed to decide if that contig can be used to merge two of the original assembly contigs, as follows (see Fig. 3a). The longest match to each of the start and end of the SPAdes contigs is identified. If these matches are to different original assembly contigs, nucmer did not report another longer match to the SPAdes contig, and the positions and orientations are such that the original contigs can be joined, then a new merged contig is constructed. By default, the matches must be at least 4000 bp long, within 1000 bp of the start or end of the SPAdes contig, and within 15,000 bp of the start or end of the original assembly contigs. More tolerance is allowed in the assembly contigs because they often start and end with a low-quality sequence. When a join is made, the filtered reads are remapped to the new merged assembly and the process of read filtering, assembly, and contig merging is repeated until no more contigs can be merged.

#### Circularization

Once all possible contig merges are made, the final SPAdes assembly from iterative merging is again aligned to the merged assembly using nucmer with the same settings. Circlator attempts to circularize each contig in turn. First, an attempt is made to match the contig to a SPAdes contig that was identified as circular by SPAdes. If the original assembly contig has nucmer matches that cover at least 95 % of its length, all to the same SPAdes circular contig, and one of those nucmer matches has length at least 95 % of the length of the SPAdes contig, then the original assembly contig is replaced with the SPAdes contig. If no such SPAdes contig is found, then the longest match to the start and to the end of the original contig is identified, using the same criteria as in the merging stage above. If these two matches are the same, then the second longest match is used. If the two matches are to the same SPAdes contig, and are in the correct orientations and positions (as in Fig. 3b), then the contig is circularized. If neither method works, then the original assembly contig is not changed.

#### Contig refinement

The assembly is refined further by discarding all contigs shorter than a minimum length, by default 2000 bp. Next, all non-circular contigs that are found to be redundant are removed, using the following method. The assembly is aligned to itself using nucmer with the same options as above for contig merging. Contig A is said to be contained in contig B if it has not previously been identified as circular, and there is a nucmer alignment to contig B with at least 95 % identity and of length at least 95 % of the length of contig A. All such instances of contained contigs are identified, and the relationships are expanded using transitivity. For example, if A is contained in B and B is contained in C, then we ensure that A is contained in C (in case nucmer did not report that A is contained in C). For each set of equivalent contigs, specifically where every contig of the set is contained in all other contigs in the set, only the longest contig of the set is retained. Finally, each remaining contig that is contained in another contig is removed from the assembly.

The last stage of the pipeline rearranges all circular contigs to begin at a known gene. By default each contig is searched against the nucleotide sequences of 162 *dnaA* genes obtained from RefSeq [25] (see Additional file 1 for details). The user can provide any alternative FASTA file of genes, but *dnaA* is used by default because this gene is found in most bacteria and is usually close to the origin of replication [26]. The search is performed on translated nucleotide sequences using the PROmer algorithm of MUMmer, and hits with a minimum percentage identity of 80 are retained using delta-filter -i 80. The first match to the entire length of any *dnaA* gene is used to rearrange the contig so that it starts with that gene on its forward strand. If no such match is found, the start of the gene nearest to the middle of the contig is used instead, identified using Prodigal [27] with the option -c to prevent prediction at contig ends.

### Optimizing existing methods

In addition to developing Circlator, two existing methods were modified and automated, for comparison with Circlator. The modifications were made to improve the effectiveness and accuracy of these methods at circularizing assemblies.

#### BLAST method

One approach to solving the circularization problem is to use BLAST to identify a sequence that is in common at the start and end of a contig. This has been implemented in a script called check_circularity.pl included with the SPRAI assembler. Whenever an overlap is found between the start and end of a contig, it is identified as circular and the duplicated sequence at the start of the contig is removed. A circular sequence that is shorter than twice the read length is often assembled into a contig consisting of multiple tandem copies of the original sequence; for example, see Fig. 1c. In this situation, trimming one duplicated sequence is not sufficient to fix the contig because multiple copies of the true sequence remain. To solve this, we developed a script (sprai_check_circularity_iterative.py, included in Additional file 3) that iteratively runs the check_circularity.pl script until no more sequence can be removed from any contig ends. The check_circularity.pl script included in SPRAI version 0.9.9.1 was used, which in turn ran blast+ version 2.2.30 [28].

#### Minimus2 method

The protocol recommended by PacBio to identify and repair circular contigs [29] is based on using Minimus2. A copy of this protocol is included in Additional file 1. We automated this manual protocol, with the improvements described below, and added it as an option to Circlator. Minimus2 from version 3.1.0 of the AMOS suite was used. First, Minimus2 is run on the input assembly to merge any overlapping contigs (this is optional, and not part of the original protocol). An attempt is then made to circularize each contig in turn (see Additional file 1: Figure S34) by breaking it in half to make two smaller contigs, and using the two smaller contigs as input to Minimus2. If the original contig had a sequence in common between its start and end, then this should be recognized by Minimus2 and the two smaller contigs will be merged into a single circularized contig. If Minimus2 outputs one contig then this contig is used, otherwise the original, unbroken, contig is kept. The original protocol involved breaking every contig in half and running Minimus2 once on all of the broken contigs. We treat each contig separately because Minimus2 can incorrectly merge parts of different contigs when it is run on all the contig halves pooled together. Moreover, Minimus2 often fails to run, in which case the original contig is retained, whereas the original protocol would produce no output.

### Circlator software

Circlator is open source and available for Linux at http://sanger-pathogens.github.io/circlator/ under the GPLv3 license. For this study, we used Circlator version 0.14.0, which depended on using SPAdes version 3.5.0, MUMmer version 3.23, BWA-MEM version 0.7.12, Prodigal version 2.60, and SAMtools [30] version 0.1.9. It has low memory usage and a short run time (see Supplementary text, Additional file 2: Table S7, Additional file 1: Table S8, and Additional file 1: Figure S35). Circlator is easy to use, with a single call required to run the whole pipeline, and is also modular, so that any stage of the pipeline can be run in isolation.

### Data availability

The NCTC reads are available from the European Nucleotide Archive with the following accession numbers: NCTC3610 (ERR581147, ERR581145), NCTC10005 (ERR688913, ERR688954), NCTC10833 (ERR879369), NCTC10963 (ERR710263), NCTC11192 (ERR832407), NCTC12419 (ERR657651, ERR657671), NCTC13251 (ERR768071), NCTC13277 (ERR879377), NCTC13307 (ERR550486, ERR550480, ERR581143), NCTC13348 (ERR550498, ERR550489), NCTC13349 (ERR772449), NCTC13360 (ERR879378), NCTC13616 (ERR879380, ERR902071), and NCTC13626 (ERR879381, ERR902070).

All *P. falciparum* reads are available from the ENA. The PacBio reads have accessions ERR951787 to ERR951797 inclusive. The improved apicoplast sequence was generated using 454 reads with accessions ERR102953–4 and Illumina reads with accessions ERR007655–6.

The *H. sapiens* reads are available from the ENA, with accession numbers SRR1304331 to SRR1304530 inclusive.

The ONT data used in this study were downloaded from http://wgs-assembler.sourceforge.net/wiki/index.php/PBcR#Assembling_a_MinION_dataset. The original data are available under accession numbers ERX708228 to ERX708231 inclusive.

## Ethical approval

No ethical approval was required for this study.

## Additional files

Additional file 1
**Supplementary text and figures supporting the main text.** (PDF 803 kb)

Additional file 2
**An Excel spreadsheet containing Tables S1–5 and S7.** **Table S1.** NCTC project IDs and accession numbers for the PacBio bacterial samples **Table S2.** A complete breakdown of results on the PacBio bacterial samples (an expanded version of Table 1) **Table S3.** QUAST results on the PacBio bacterial samples **Table S4.** Results of testing user-defined parameters for all NCTC data sets **Table S5.** Results of testing user-defined parameters for the MinION data set **Table S7.** Run time and memory usage for all data sets (excluding those in Tables S4 and S5). (XLS 305 kb)

Additional file 3 A gzipped archive containing all scripts, corrected reads, assemblies, and other data necessary to reproduce the results. (gzipped archive 11GB)

## Abbreviations

NCTC: National Collection of Type Cultures
ONT: Oxford Nanopore Technologies
PacBio: Pacific Biosciences

## References

[1] Staden R (1979). A strategy of DNA sequencing employing computer programs. Nucleic Acids Res.

[2] Koren S, Phillippy AM (2015). One chromosome, one contig: complete microbial genomes from long-read sequencing and assembly. Curr Opin Microbiol.

[3] Chin CS, Alexander DH, Marks P, Klammer AA, Drake J, Heiner C (2013). Nonhybrid, finished microbial genome assemblies from long-read SMRT sequencing data. Nat Methods.

[4] Berlin K, Koren S, Chin CS, Drake JP, Landolin JM, Phillippy AM (2015). Assembling large genomes with single-molecule sequencing and locality-sensitive hashing. Nat Biotechnol.

[5] SPRAI: Single pass read accuracy improver. http://zombie.cb.k.u-tokyo.ac.jp/sprai/index.html. Accessed 19 Nov 2014.

[6] Eyre DW, Golubchik T, Gordon NC, Bowden R, Piazza P, Batty EM (2012). A pilot study of rapid benchtop sequencing of *Staphylococcus aureus* and *Clostridium difficile* for outbreak detection and surveillance. BMJ Open.

[7] Köser CU, Holden MTG, Ellington MJ, Carwright EJ, Brown NM, Ogilvy-Stuart AL (2013). Rapid whole-genome sequencing for investigation of a neonatal MRSA outbreak. N Engl J Med.

[8] Shao L, Martin MV, Watson SJ, Schatzberg A, Akil H, Myers RM (2008). Mitochondrial involvement in psychiatric disorders. Ann Med.

[9] Cai N, Chang S, Li Y, Li Q, Hu J, Liang J (2015). Molecular signatures of major depression. Curr Biol.

[10] Hudson G, Carelli V, Spruijt L, Gerards M, Mowbray C, Achilli A (2007). Clinical expression of Leber hereditary optic neuropathy is affected by the mitochondrial DNA-haplogroup background. Am J Hum Genet.

[11] Hao H, Bonilla E, Manfredi G, DiMauro S, Moraes CT (1995). Segregation patterns of a novel mutation in the mitochondrial tRNA glutamic acid gene associated with myopathy and diabetes mellitus. Am J Hum Genet.

[12] Altschul SF, Gish W, Miller W, Myers EW, Lipman DJ (1990). Basic local alignment search tool. J Mol Biol.

[13] Sommer DD, Delcher AL, Salzberg SL, Pop M (2007). Minimus: a fast, lightweight genome assembler. BMC Bioinformatics.

[14] NCTC 3000 Project. https://www.phe-culturecollections.org.uk/collections/nctc-3000-project.aspx. Accessed 28 Jul 2015.

[15] Loman NJ, Quick J, Simpson JT (2015). A complete bacterial genome assembled de novo using only nanopore sequencing data. Nat Methods.

[16] Gurevich A, Saveliev V, Vyahhi N, Tesler G (2013). QUAST: quality assessment tool for genome assemblies. Bioinformatics.

[17] Salzberg SL, Phillippy AM, Zimin A, Puiu D, Magoc T, Koren S (2012). GAGE: a critical evaluation of genome assemblies and assembly algorithms. Genome Res.

[18] Gardner MJ, Hall N, Fung E, White O, Berriman M, Hyman RW (2002). Genome sequence of the human malaria parasite *Plasmodium falciparum*. Nature.

[19] Li H. Aligning sequence reads, clone sequences and assembly contigs with BWA-MEM. arXiv. 2013. doi:arXiv:1303.3997v2[q-bio.GN].

[20] Bankevich A, Nurk S, Antipov D, Gurevich AA, Dvorkin M, Kulikov AS (2012). SPAdes: a new genome assembly algorithm and its applications to single-cell sequencing. J Comput Biol: J Comput Mol Cell Biol..

[21] Loman NJ, Quinlan AR (2014). Poretools: a toolkit for analyzing nanopore sequence data. Bioinformatics.

[22] PBcR Assembler. http://wgs-assembler.sourceforge.net/wiki/index.php/PBcR#Assembling_a_MinION_dataset. Accessed 22 Apr 2015.

[23] Kurtz S, Phillippy A, Delcher AL, Smoot M, Shumway M, Antonescu C (2004). Versatile and open software for comparing large genomes. Genome Biol.

[24] Carver T, Harris SR, Berriman M, Parkhill J, McQuillan JA (2012). Artemis: an integrated platform for visualization and analysis of high-throughput sequence-based experimental data. Bioinformatics.

[25] Tatusova T, Ciufo S, Fedorov B, O’Neill K, Tolstoy I (2014). RefSeq microbial genomes database: new representation and annotation strategy. Nucleic Acids Res.

[26] Mackiewicz P, Zakrzewska-Czerwińska J, Zawilak A, Dudek MR, Cebrat S (2004). Where does bacterial replication start? Rules for predicting the oriC region. Nucleic Acids Res.

[27] Hyatt D, Chen GL, Locascio PF, Land ML, Larimer FW, Hauser LJ (2010). Prodigal: prokaryotic gene recognition and translation initiation site identification. BMC Bioinformatics.

[28] Camacho C, Coulouris G, Avagyan V, Ma N, Papadopoulos J (2009). BLAST+: architecture and applications. BMC Bioinformatics.

[29] PacBio circularizing and trimming. https://github.com/PacificBiosciences/Bioinformatics-Training/wiki/Circularizing-and-trimming. Accessed 22 Jun 2015.

[30] Li H, Handsaker B, Wysoker A, Fennell T, Ruan J, Homer N (2009). The sequence alignment/map format and SAMtools. Bioinformatics.

[31] Krzywinski M, Schein J, Birol I, Connors J, Gascoyne R, Horsman D (2009). Circos: an information aesthetic for comparative genomics. Genome Res.

