# Supplementary material for: Circlator: automated circularization of genome assemblies using long sequencing reads
Source: Genome Biol. 2015 Dec 29;16:294. doi: 10.1186/s13059-015-0849-0 (PMC4699355; doi:10.1186/s13059-015-0849-0)
Supplement: Additional file 1 — Supplementary text and figures supporting the main text. (PDF 803 kb) [file 13059_2015_849_MOESM1_ESM.pdf]

Supplementary Material of:

Circlator: automated circularization of genome assemblies using long sequencing reads

Martin Hunt [1], Nishadi De Silva [1], Thomas D Otto [1], Julian Parkhill [1], Jacqueline A Keane [1] and Simon R Harris [1]

[1]Wellcome Trust Sanger Institute, Wellcome Trust Genome Campus, Cambridge, CB10 1SA, UK

1 Reference dnaA genes

The panel of reference dnaA genes was produced as follows. The term:
`'dnaA[sym] AND ("srcdb refseq"[Properties] AND alive[property])'`
was used to search the 'gene' database at the NCBI website `http://www.ncbi.nlm.nih.gov/`. All the results were saved as a 'Tabular (text)' file, which was used as input to the script `get_dnaA.pl`. This script downloads the sequences, and retains only those that are in the length range 1000-1600bp, begin with a start codon and end with a stop codon.

2 PacBio circularization protocol

The following is a verbatim copy of the PacBio circaulariztion protocol, obtained from `https://github.com/PacificBiosciences/Bioinformatics-Training/wiki/Circularizing-and-trimming` on 22nd June 2015.

1. Open up the `polished_assembly.fasta` file in a text editor.
2. In the middle of the circular contig introduce a break, i.e. a new line '`>Break`'. It does not matter where in the sequence you introduce the break, but the sequence immediately after the break will be the start of your circularized sequence.
3. `toAmos -s polished_assembly.fasta -o circularized.afg`
4. `minimus2 circularized`
5. Minimus will overlap and join the ends of the two contigs, the resulting `circularized.fasta` file should contain one contig for the sequence in which you introduced a break.
6. Any sequence that was not circularized, or extra contigs in which you did not introduce a break will be in `circularized.singletons.seq`, and can be added back to the `circularized.fasta` file
   `cat circularized.singletons.seq >> circularized.fasta`
7. The sequence that was overlapped now needs to be quiver corrected, to do this simply import the circularized.fasta file into SMRT Portal as a reference and run a resequencing job with the raw data. Problems with the coverage in the region which was overlapped could indicate an issue with the circularization and the possibility that the molecule was not circular, or was split at a repeat that has not been accounted for.

3 *Plasmodium falciparum* apicoplast

To improve the *P. falciparum* 3D7 reference sequence we sequenced the genome with Illumina short insert (accessions ERR007655-6) and 454 8kb insert libraries (accessions ERR102953-4). Read coverage analysis of the Illumina data showed that the last 5kb of the apicoplast was duplicated. The duplication contained fewer than

five heterozygous SNPs, indicating that the two copies are nearly identical. With the help of the 454 large fragment library, we manually arranged the two copies as an inverted repeat separated with 16 unique bases. Further we confirmed that the plastid is circular, as expected. Previous attempts to circularize the apicoplast and confirm the orientation of the unique sequence by PCR were unsuccessful.

We used the 454 reads to confirm the overall structure of the new, circularized, sequence made by Circlator. The reads were mapped to the complete HGAP assembly using SMALT (`https://www.sanger.ac.uk/resources/software/smalt/`) version 0.7.0.1 with the options `-x -r 1 -i 13000 -y 0.8` and reads, plus their mates, that mapped to the HGAP contig corresponding to the apicoplast were retained. These reads were mapped independently to the manually created reference sequence and to the output of Circlator using SMALT with the same settings as above. Both sequences were mapped to because they have different start positions, allowing the complete circular sequence to be verified. See Supplementary Figure S21, showing the reads mapped to both sequences, confirming the boundaries of the inverted repeat sequence and the point at which Circlator rearranged the start point of the sequence.

4 Nanopore data

The Nanopore data were downloaded from the PBcR assembly wiki page (`http://wgs-assembler.sourceforge.net/wiki/index.php/PBcR#Assembling_a_MinION_dataset`), which included the 2D reads already converted to FASTQ format using poretools. This FASTQ file was used as input to the PBcR assembler, to generate the assembly and corrected reads used for this manuscript.

The original data are available under accession numbers ERX708228 to ERX708231 inclusive. Each run was converted to FASTQ format using poretools version 0.5.1 with the options `fastq --type 2D` on each of the four flowcells, to produce the FASTQ file included in the download from the PBcR wiki page.

5 Comparing assemblies and reference genomes

Supplementary Figures S2–S18 show the input assembly compared to the reference sequence, and the reference sequence compared to the output of the BLAST-based method, Circlator, and Minimus2. These figures were generated using the supplementary script `act_cartoon.py`. In each figure, the top genome is the input assembly, the middle genome is the reference, and the bottom assembly is the output of one of the three circularization methods.

Nucmer matches are shown between the genomes in blue and pink. Hits on the same strand are shown in blue, and those on opposite strands are coloured pink. All matches are shown that have a minimum identity of 98% and are either at least 5000bp long or at least 3% of the length of the input or output assembly contig length. The only exception is the nanopore data in Supplementary Figure S17, where the minimum identity was reduced to 85%.

The input assembly contigs that were merged are coloured yellow (this only applies to Circlator and the Minimus2-based method). Input assembly contigs that

were removed are coloured red (this only applies to Circlator). Any output assembly contig flagged as circular by the tool that made it is coloured green (applicable to all three tools).

The reference genome has three plots. The top and bottom plots (colored black) show the number of `nucmer` matches between the reference and the input and output assemblies. They are normalised so that they have the same y axis scale, to allow comparison. The upper plot is generated from hits to the input assembly, and the lower plot is generated from hits to the output assembly. These plots can be used to identify overlapping sequence that is removed during the circularization process. For example, on sample NCTC13616 Circlator and the BLAST-based method removed one copy of the repetitive sequence at the start and end of the input assembly contig (see Supplementary Figure S14).

The centre plot (in blue) marks repeats in the reference genome. A region is counted as a repeat if it has a `nucmer` hit to elsewhere in the reference genome. Hits must be at least 250bp long and have a percent identity of at least 98. These hits frequently match assembly contig ends, confirming that contigs often end with repeat sequences. See for example Supplementary Figure S9.

6 Evaluation of user-defined parameters

Eight key parameters were tested for a range of values on each of the NCTC sample datasets and on the MinION dataset. The ranges used and the effects on merging and circularization are given in Supplementary Tables S4 and S5, and summarized in Supplementary Table S6 and Supplementary Figures S22-S29.

The first parameter tested was `--b2r_length_cutoff`, which determines the reads that are used for reassembly of contig ends. The default value of 100,000 means that all reads mapped to contigs of length up to 100,000bp are retained. Reads mapping within 50,000bp (half of 100,000) of contigs longer than 100,000bp are also retained. This parameter was found to have the greatest effect on contig merging and circularization.

The next four parameters were options used when running `nucmer` to compare the reassembled contigs to the original assembly contigs. These were:

- `--merge_breaklen` – this is the `-b|breaklen` option of `nucmer`, decribed in the help as: ‘Set the distance an alignment extension will attempt to extend poor scoring regions before giving up’;
- `--merge_diagdiff` – this is the `-D|diagdiff` option of `nucmer`, described in the help as: ‘Set the maximum diagonal difference between two adjacent anchors in a cluster’;
- `--merge_min_id` – the minimum percent identity required to report a match (the `-i` option of `delta-filter`);
- `--merge_min_length` – the minimum length of match to report (the `-l` option of `delta-filter`).

The final three parameters related to match lengths and their distance from contig ends:

- --merge_min_length_merge – this is the minimum length of nucmer match used when circularizing a contig;
- --merge_reassemble_end – this is the maximum allowed distance between a nucmer hit and the end of a reassembly contig;
- --merge_ref_end – this is the maximum allowed distance between a nucmer hit and the end of an input assembly contig.

Seven of the 14 NCTC samples were affected for at least one Circlator run when one of these parameters were changed, usually only when extreme values were used. The effects are described below for each of the seven affected samples.

NCTC10005

Depending on the value of --b2r_length_cutoff, the chromosome was circularized in 5 of the 20 runs. When the circularization was not successful, the SPAdes contig that should have been used to circularize the chromosome contained a spurious segment of genome that belonged elsewhere. This extra segment caused Circlator to not recognise the chromosome as circular. See Supplementary Figure S30 for an example.

NCTC10833

Circularization of the chromosome was affected by --b2r_length_cutoff. The SPAdes assemblies failed for 10,000 and 20,000. The SPAdes assemblies were successful for 30,000, 40,000, 50,000, 60,000, but circularization failed. The HGAP assembly of the chromosome starts and ends with a repeat of length approximately 33,000 that is present in the middle of the chromosome. Small values of --b2r_length_cutoff meant that unique sequence flanking the repeat was not assembled, so Circlator could not circularize the sequence. It rejected the matches between the SPAdes contig and HGAP contig ends, because there was a longer match to elsewhere. Circularization was successful if --b2r_length_cutoff was at least 70,000, meaning that the end 35,000 bases of each contig is reassembled with SPAdes, because the hit to the repeat in the middle of the chromosome was shorter than the hits to the end of the chromosome. See Supplementary Figure S31 for ACT screenshots comparing the SPAdes and HGAP assemblies where circularization was not successful.

NCTC10963

The SPAdes assembly of this sample failed when --b2r_length_cutoff was set to 10,000. No merges were made for all other tested values of --b2r_length_cutoff, except 30,000. With this value, SPAdes produced a contig that could be used to merge two small input HGAP assembly contigs. These two small input contigs were not reassembled by SPAdes with all larger values of --b2r_length_cutoff, we presume because they had relatively low read coverage (approximately 3X) compared to the chromosome (approximately 12X).

Circlator option	NCTC Default*	Range tested	Effect on merging and circularization
`--b2r_length_cutoff`	100000	10000-200000	Various changes
`--merge_breaklen`	500	100-1500	Large values needed for circularization (MinION only)
`--merge_diagdiff`	25	5-50	None
`--merge_min_id`	95	70-100	Must be: $\leq$ 95 (NCTC), or $\leq$ 85 (MinION)
`--merge_min_length`	500	100-2000	With large values, circularization affected for one NCTC sample
`--merge_min_length_merge`	4000	500-10000	With small/large values, circularization affected for two NCTC samples
`--merge_reassemble_end`	1000	200-3000	With small values, merging affected for one NCTC sample
`--merge_ref_end`	15000	1000-30000	With small/large values, circularization affected for NCTC two samples

*Defaults for MinION were the same, except `--merge_breaklen 1000` and `--merge_min_id 85`.

Table S6: Summary of user-defined options that were tested, and the effects on the NCTC and MinION datasets.

NCTC13307

Two extra contig merges were made with `--b2r_length_cutoff 30000`, because of differences in the SPAdes assembly. With a value of 30000, the SPAdes assembly contained contigs that allowed two joins of HGAP contigs, but values larger than 30000 resulted in different SPAdes assemblies that meant Circlator could not make these joins. See Supplementary Figure S32 for an ACT screenshots comparing the SPAdes and HGAP assemblies when `--b2r_length_cutoff 30000` and `40000`, for one of the two joins.

The option `--merge_reassemble_end` with values 200, 400 and 600 resulted in an extra contig merge. This option is the maximum distance allowed for from the end of a SPAdes contig, for a `nucmer` hit to be used for merging contigs. All values greater than 600 did not produce the extra merge, because of another hit that was within 800bp of a contig end. The non-uniqueness of the hits prevented Circlator from making the merge.

NCTC13348

On this sample, Circlator made one extra merge with `--b2r_length_cutoff 30000`, due to differences in the SPAdes assemblies. With the value of 30000, the assembly was more contiguous and contained a contig that enabled the extra merge. In turn, this extra merge meant that the chromosome was in one piece, and could then be circularized.

Using `--b2r_length_cutoff 20000` resulted in a false circularization, because SPAdes produced a contig that was an assembly of just the start and end of an input HGAP contig. Larger values of `--b2r_length_cutoff` resulted in SPAdes contigs

that did not contain this error, so that that Circlator did not falsely circularize the contig.

Using values greater than 1500 for `--merge_min_length_merge`, which includes the default of 4000, resulted in one missed circularization. One contig was circularized for values of 500, 1000 and 1500 because one of the `nucmer` matches between this contig and the corresponding SPAdes contig was 1912 long. This meant that it could be used to circularize with `--merge_min_length_merge 1500` (or smaller), but not `--merge_min_length_merge 2000` (or larger).

Values of `--merge_ref_end` in the range 1000-21000 resulted in a correctly circularized contig. However, there was another `nucmer` match to this contig (not used for circularization) that was 21122bp from its end. Using values of at least 21122 for `--merge_ref_end` prevented Circularization, because Circlator then found two matches to the end of the contig, In such an ambiguous situation, Circlator does not make the circularization.

NCTC13360

`--b2r_length_cutoff 30000` resulted in one incorrect merge of two input contigs corresponding to distinct plasmids. This was caused by SPAdes producing an incorrect contig that was then used to merge these two input contigs. This assembly error did not occur for other values of `--b2r_length_cutoff`. Small values of this option (less than 40000) generally resulted in fewer merges because the contig ends were simply not assembled by SPAdes, we presume due to low read coverage.

Values of 170000, 180000, 190000 and 200000 for `--b2r_length_cutoff` resulted in two missed circularizations. These were caused by SPAdes producing a missassembled contig that consisted of the ends of both contigs.

Small values of `--merge_ref_end` resulted in fewer merges because `nucmer` hits that could have been used to make circularizations were excluded, as they were not close enough to the ends of contigs.

NCTC3610

One merge was missed using `--b2r_length_cutoff` with values less than 60000 because the SPAdes assembly was too fragmented. Similarly, differences in the SPAdes assemblies resulted in a missed circularization, also when this value was less than 60000.

Using values of 1400 and higher with `--merge_min_length` resulted in a missed circularization. This was caused by shorter `nucmer` hits being ignored, due to the cutoff.

MinION data

The same ranges of parameter values were tested on the MinION data as used on the NCTC data, but `--merge_min_id 85 --merge_breaklen 1000` were used as the default values, because these changes are required for successful circularization. Since the input assembly consisted of the chromosome assembled into one contig, the merging of contig pairs is not relevant for these data. We need only consider whether or not the chromosome was successfully circularized. As expected due to

	CPU (s)	Wall clock (s)	RAM (MB)
BLAST	2.14	7	26
Circlator	394.95	390	245
Minimus2	28.63	38	62

Table S8: Median CPU time, wall clock time and peak memory usage calculated across all datasets.

the lower accuracy of MinION data, low values of `--merge_min_id` ($\leq$ 85) and high values of `--merge_breaklen` ($\geq$ 600) were required for circularization to be successful (Supplementary Table S5).

Similarly to the NCTC data, Circlator was most sensitive to changes in the option `--b2r_length_cutoff`. The SPAdes assembly failed for values 10,000 to 40,000. Circularization was successful for values 50,000, 60,000, 80,000-120,000, 180,000 and 190,000 and was not successful for values 70,000, 130,000-170,000 and 200,000 (Supplementary Table S5).

Circularization failed with `--b2r_length_cutoff 70000` because the SPAdes reassembly was particularly fragmented. Varying this parameter resulted in SPAdes reassemblies consisting of 1-9 contigs with maximum contig lengths of approximately 39-94kbp, except for the SPAdes assembly with `--b2r_length_cutoff 70000`, which consisted of 625 contigs and a maximum contig length of 8552bp. The remaining failed circularizations were caused by differences in the SPAdes reassemblies: either one of both ends of the chromosome was not present in the SPAdes contigs, or SPAdes did not produce a suitable contig that spanned the start and end of the chromosome.

7 Run time and memory

The values used for peak memory usage were those reported by the compute farm job scheduling software Platform Load Sharing Facility (LSF). It polls the memory usage every minute and reports the maximum value when a job finishes. It also reports the total CPU time and wall clock time. The running times and memory usage are summarised in Supplementary Figure S35. Although Circlator has a longer running time than the other methods, the median time is under 7 minutes (Supplementary Tables S6 and S8). This is still relatively insignificant compared to the run time of an assembly (typically several hours, using multiple threads). The memory usage of all the tools is low, with a maximum memory of 490MB used by Circlator on sample NCTC13348.

The majority of the running time of Circlator is spent mapping the reads with BWA and running assemblies with SPAdes. Although we ran all analysis using one thread, the wall clock time could be reduced by using more threads with BWA and SPAdes via the option `--threads` to Circlator.

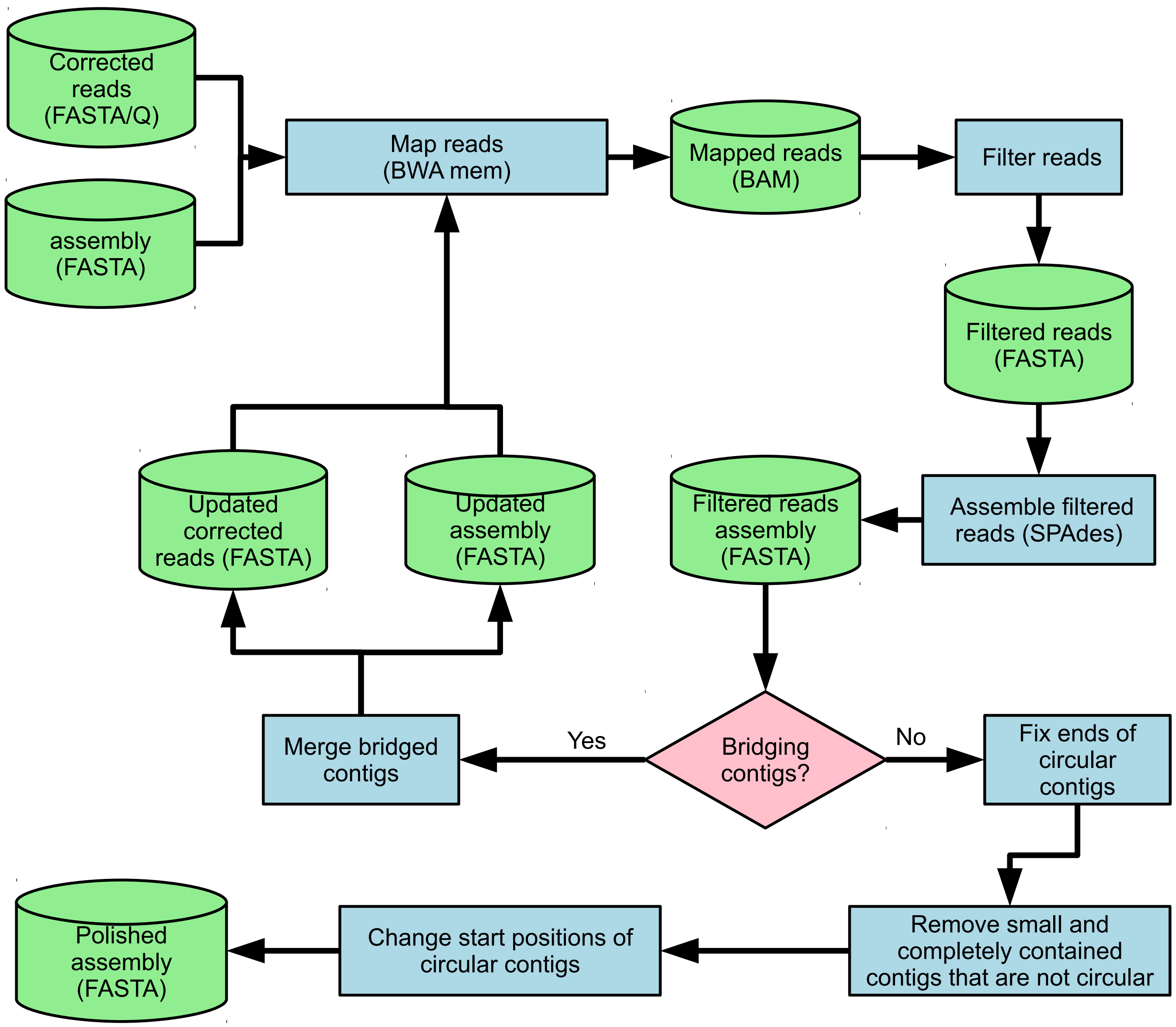

Figure S1: Workflow of Circlator

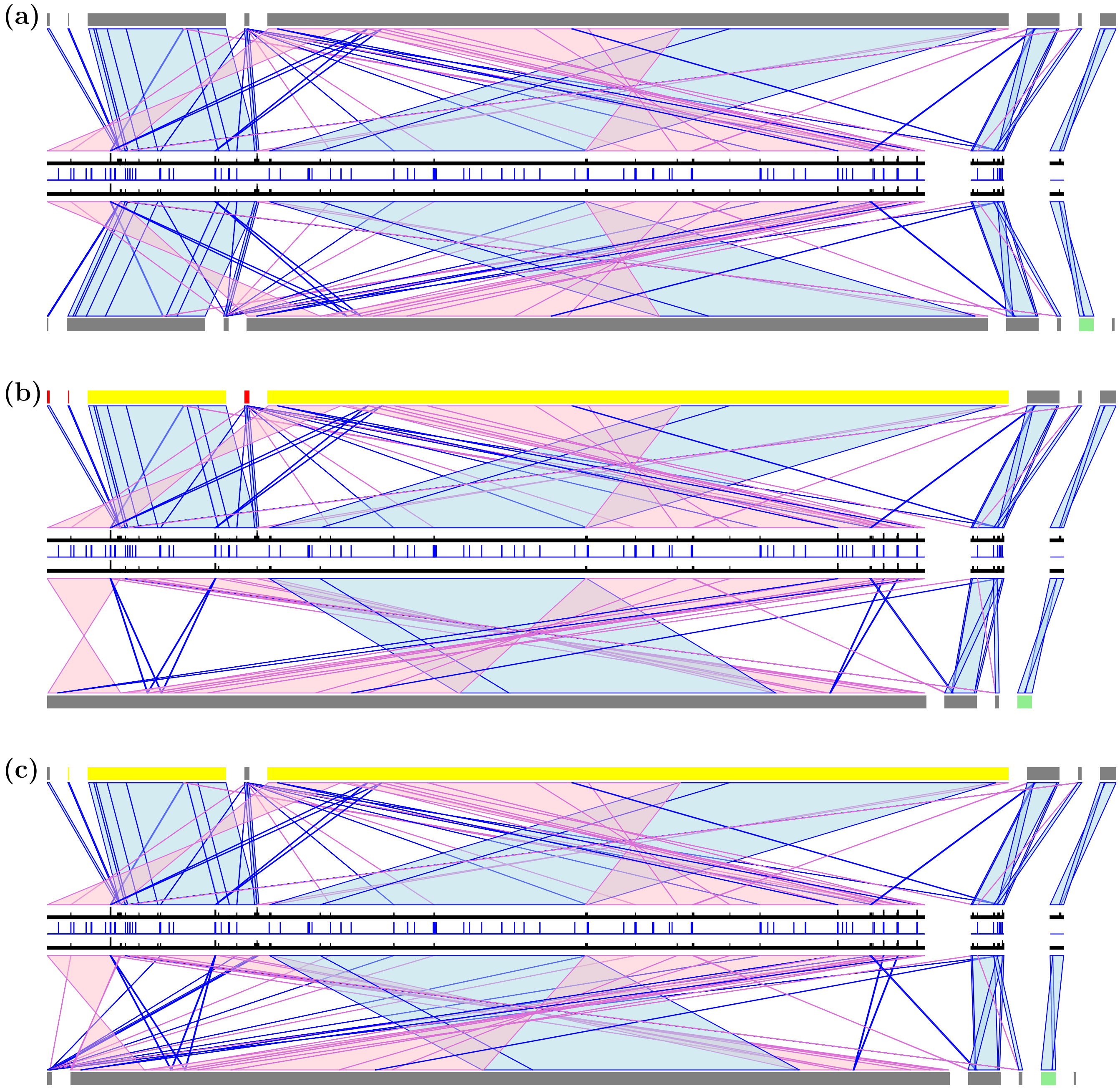

Figure S2: Comparison of circularizing NCTC sample NCTC10005 using (a) BLAST, (b) Circlator and (c) Minimus2.

(a)

(b)

(c)

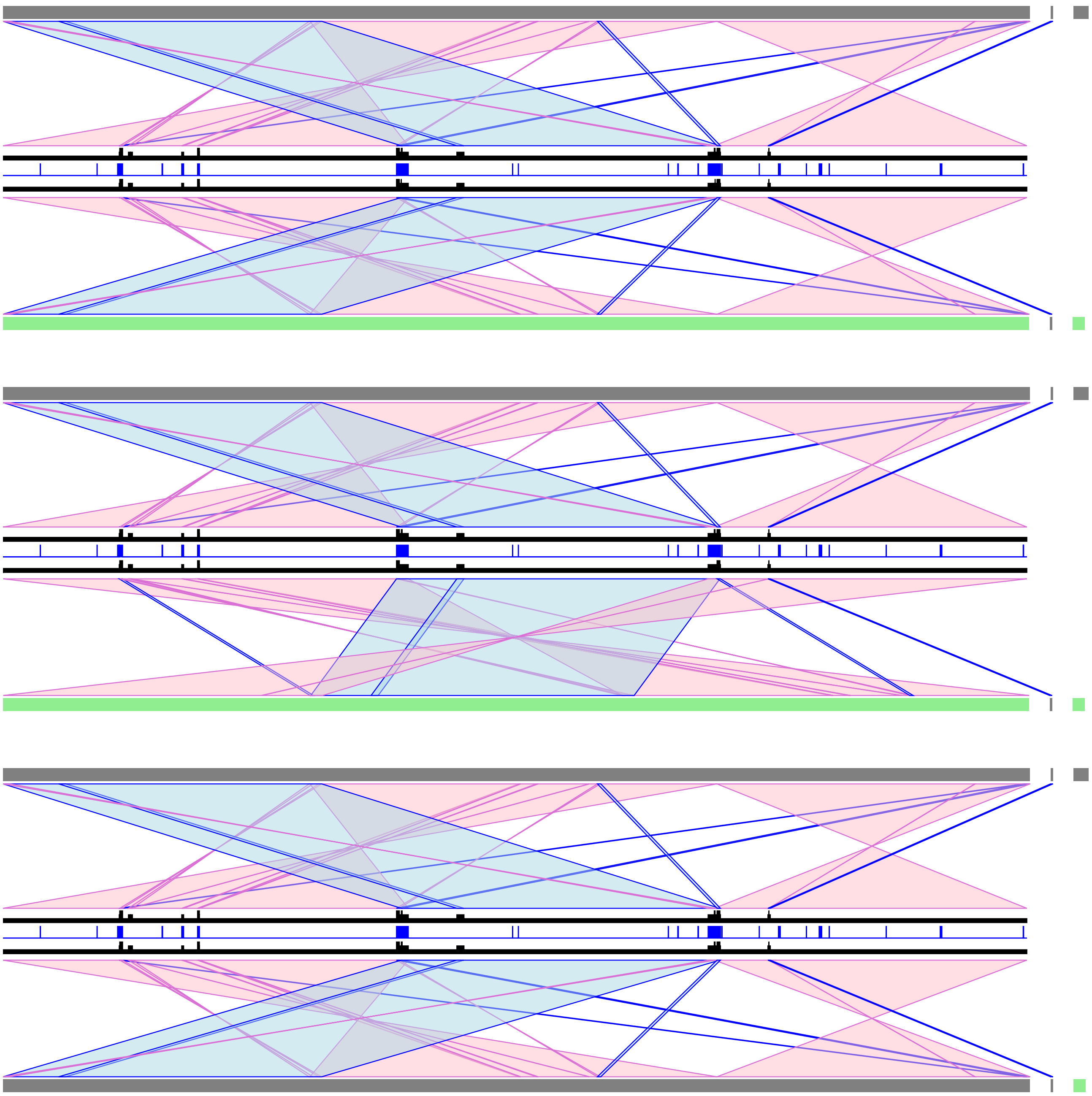

Figure S3: Comparison of circularizing NCTC sample NCTC10833 using (a) BLAST, (b) Circlator and (c) Minimus2.

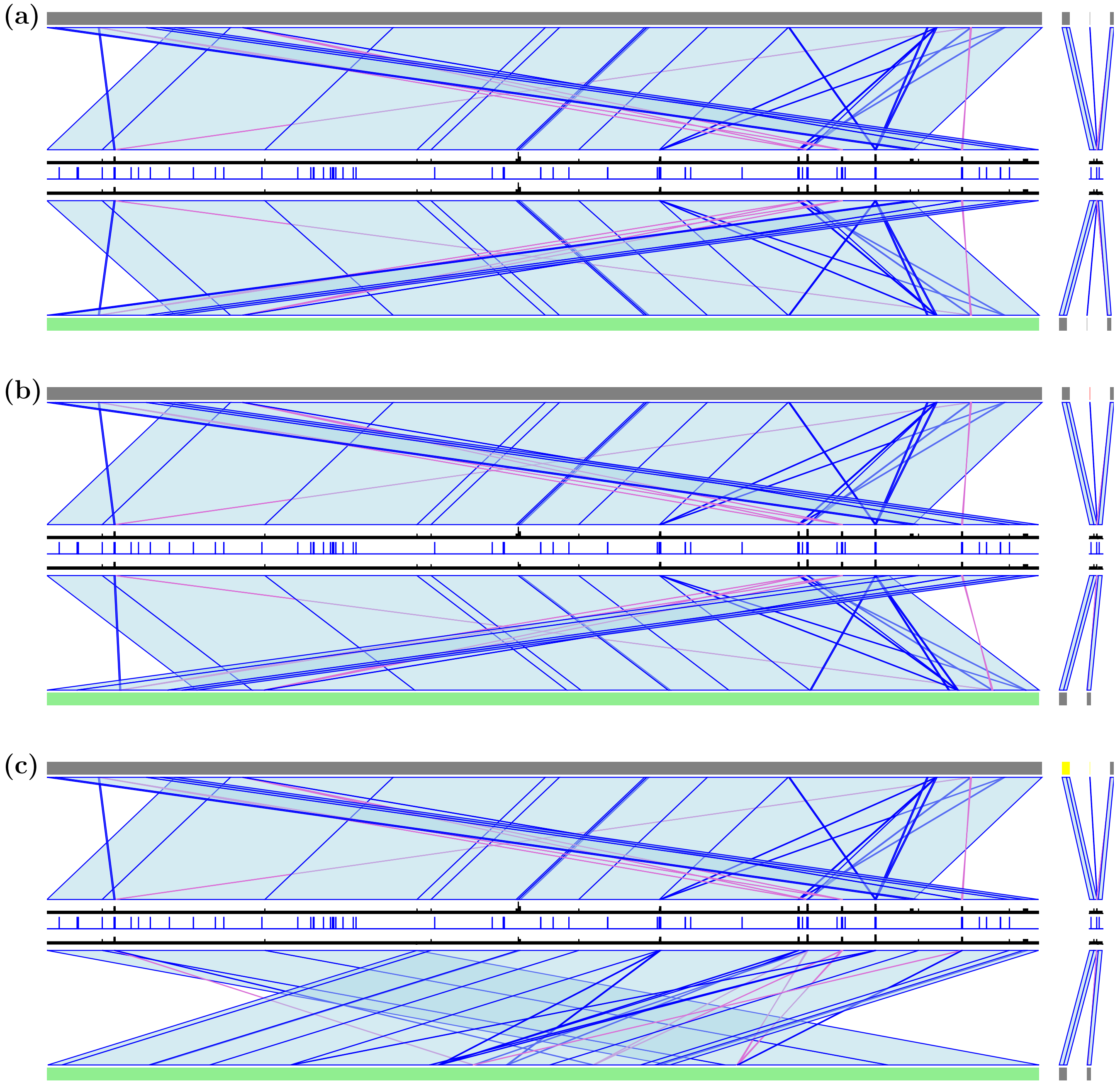

Figure S4: Comparison of circularizing NCTC sample NCTC10963 using (a) BLAST, (b) Circlator and (c) Minimus2.

(a)

(b)

(c)

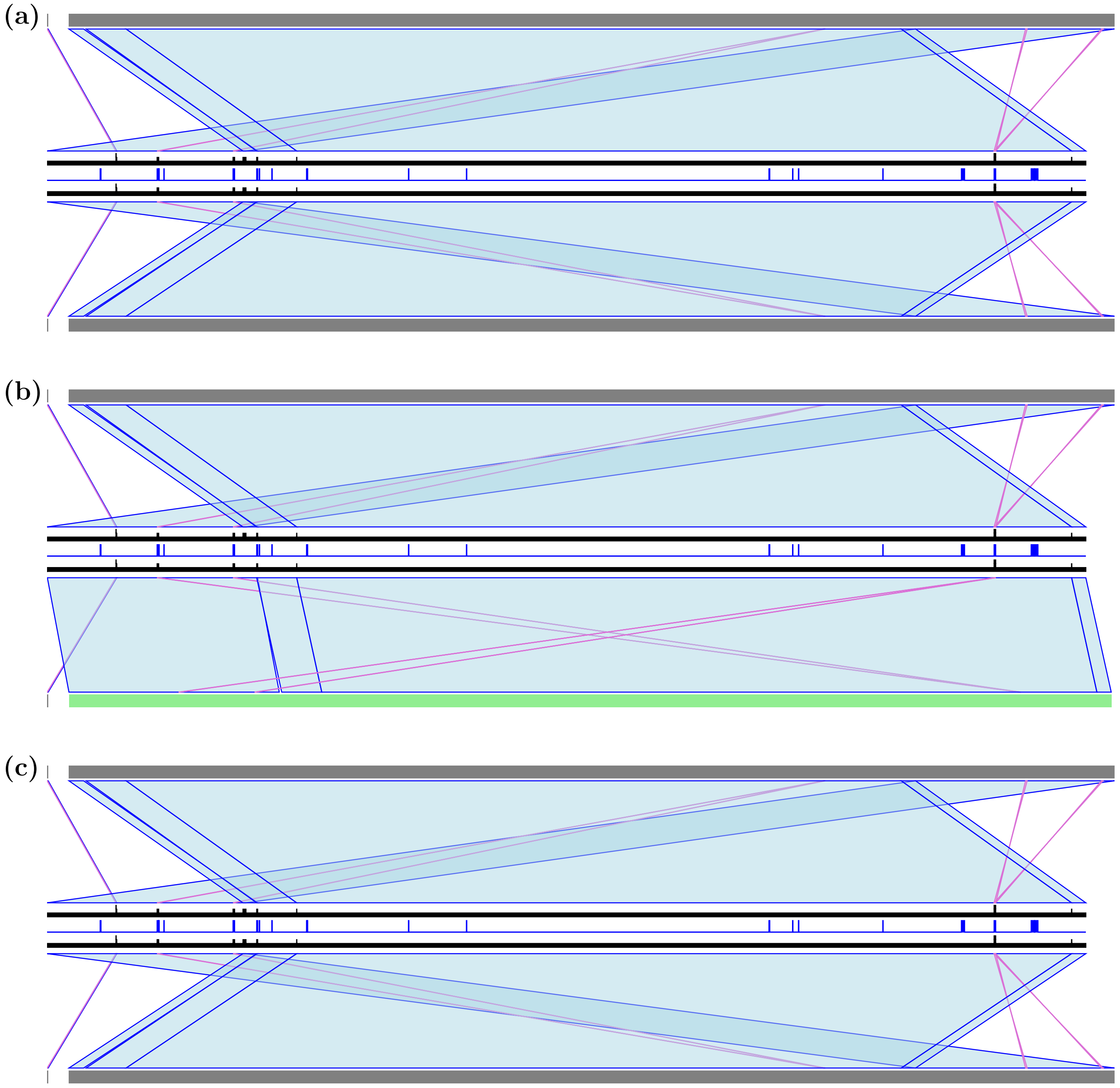

Figure S5: Comparison of circularizing NCTC sample NCTC11192 using (a) BLAST, (b) Circlator and (c) Minimus2.

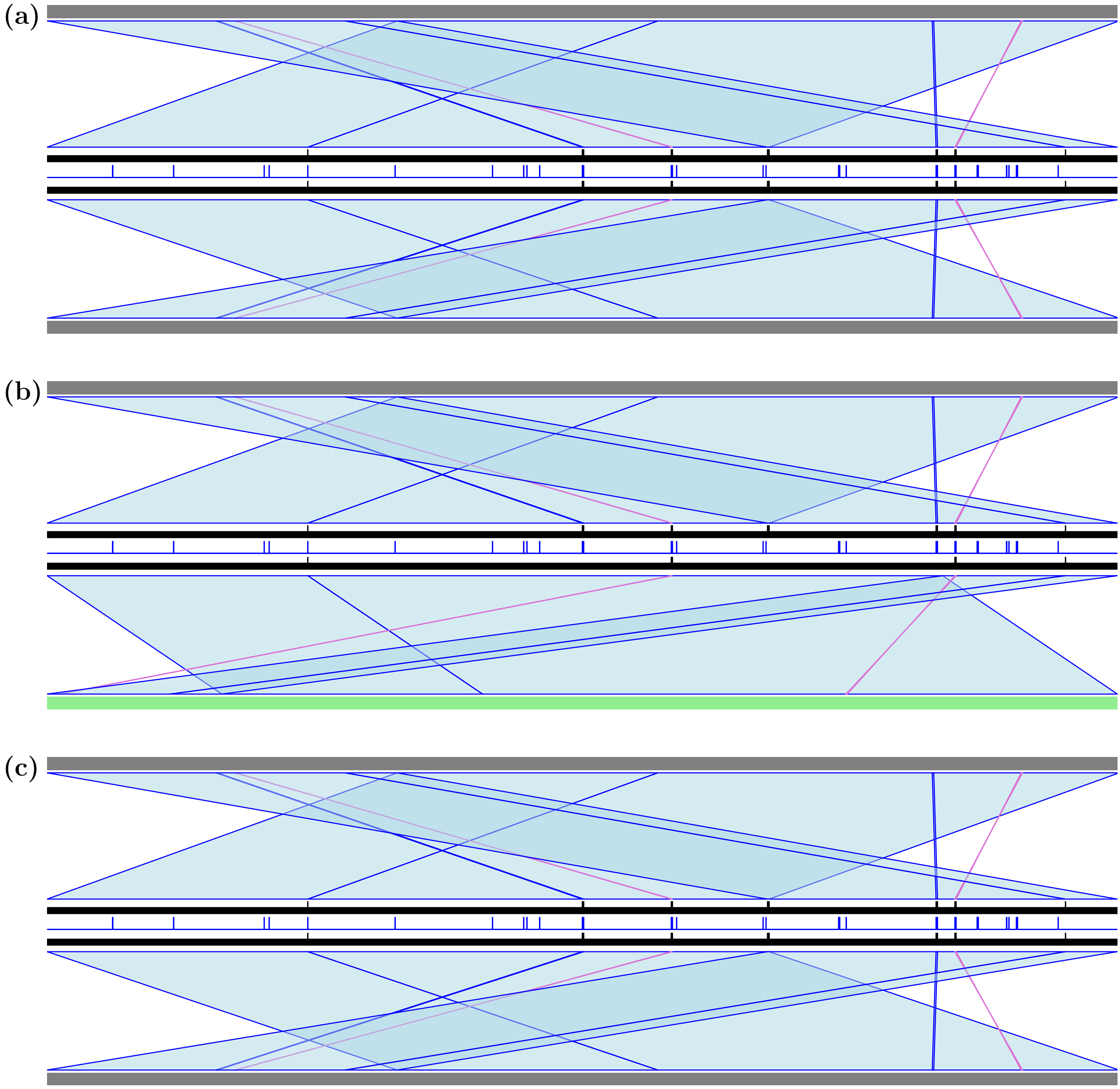

Figure S6: Comparison of circularizing NCTC sample NCTC12419 using (a) BLAST, (b) Circlator and (c) Minimus2.

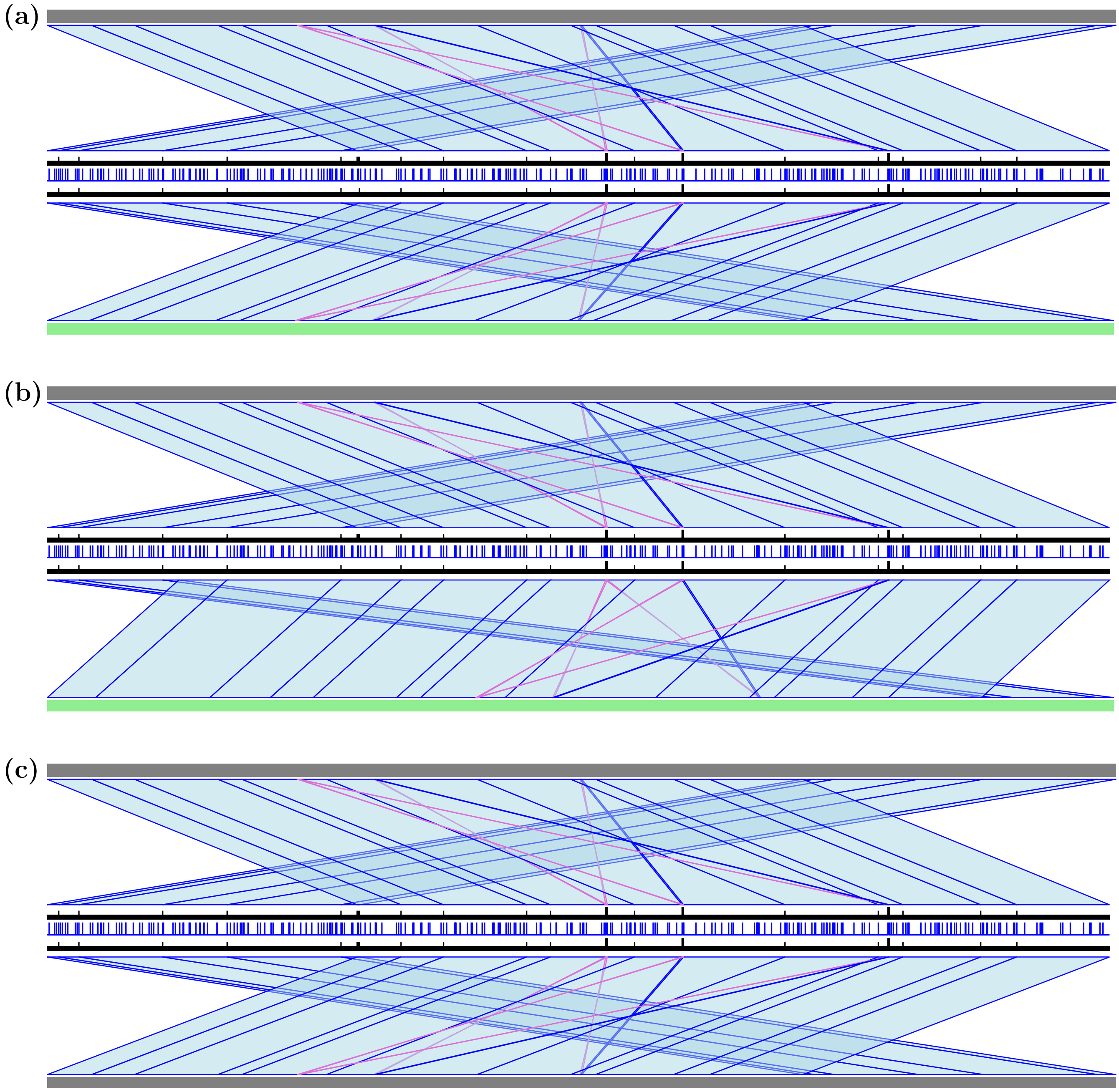

Figure S7: Comparison of circularizing NCTC sample NCTC13251 using (a) BLAST, (b) Circlator and (c) Minimus2.

(a)

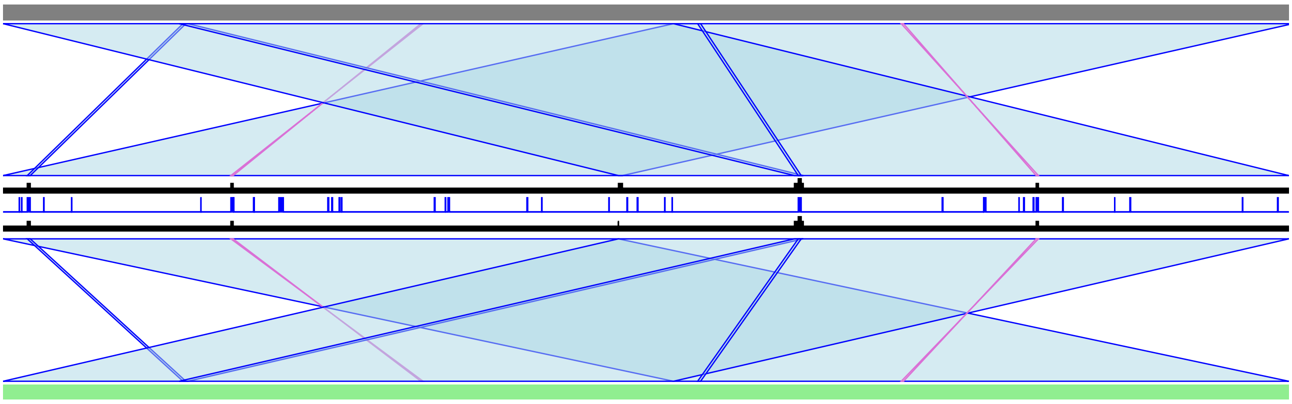

(b)

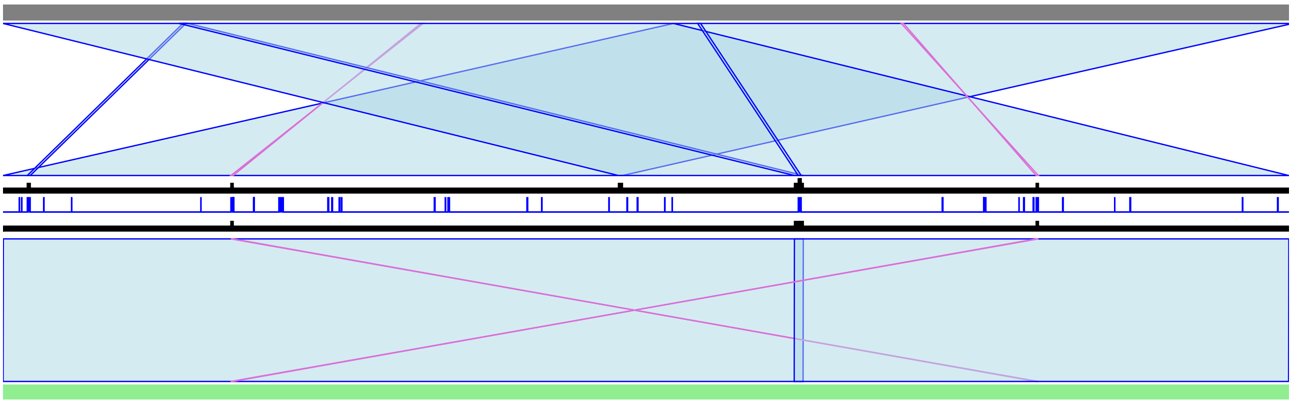

(c)

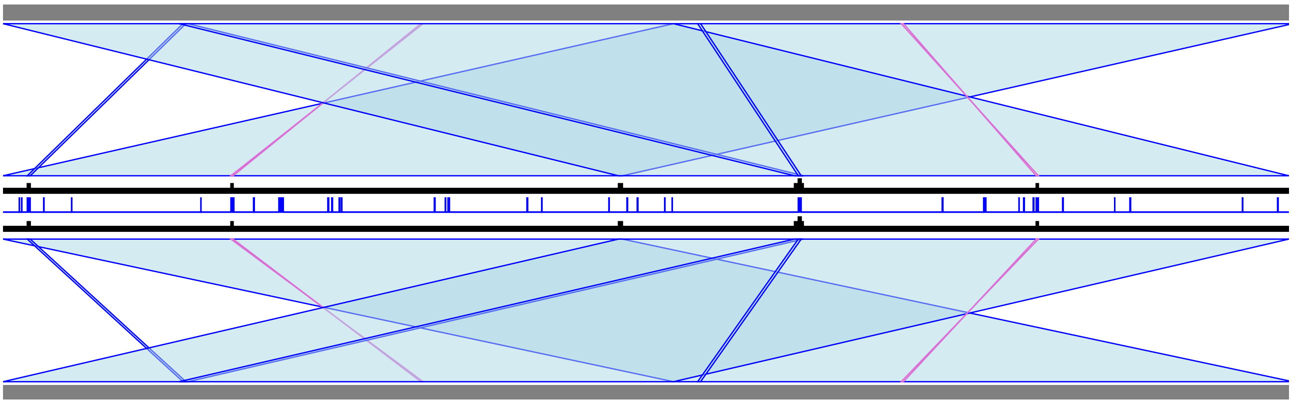

Figure S8: Comparison of circularizing NCTC sample NCTC13277 using (a) BLAST, (b) Circlator and (c) Minimus2.

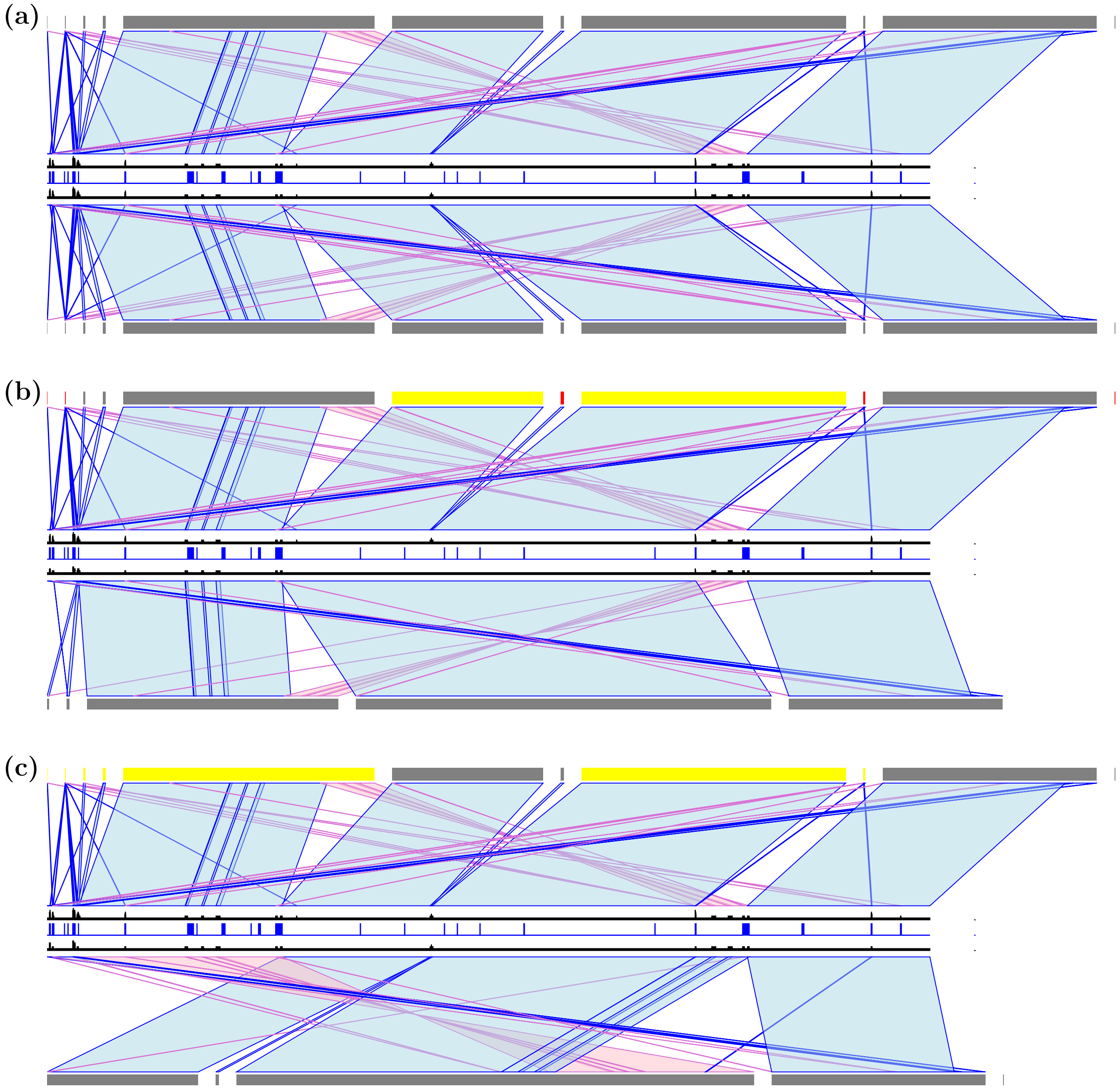

Figure S9: Comparison of circularizing NCTC sample NCTC13307 using (a) BLAST, (b) Circlator and (c) Minimus2.

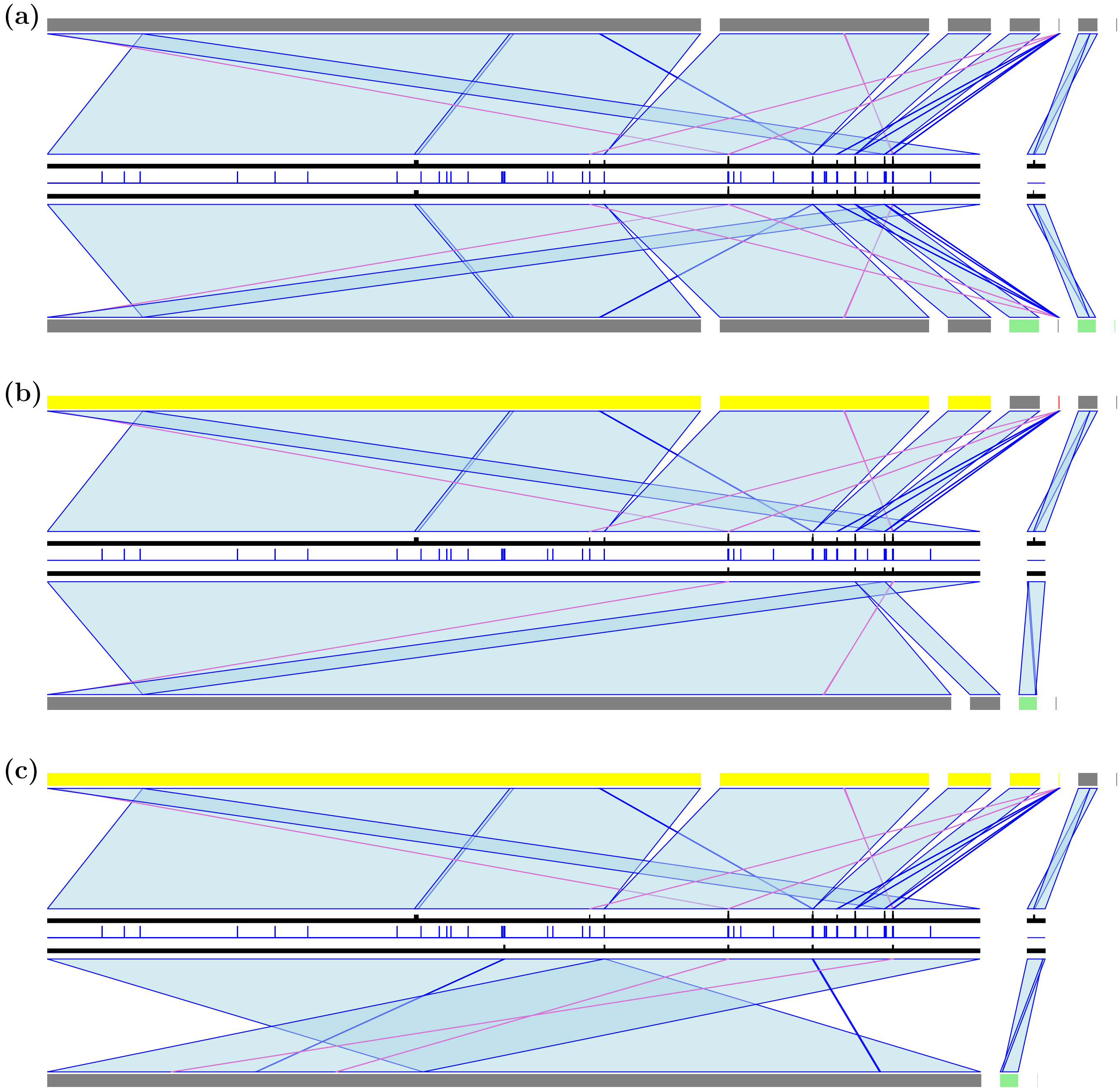

Figure S10: Comparison of circularizing NCTC sample NCTC13348 using (a) BLAST, (b) Circlator and (c) Minimus2.

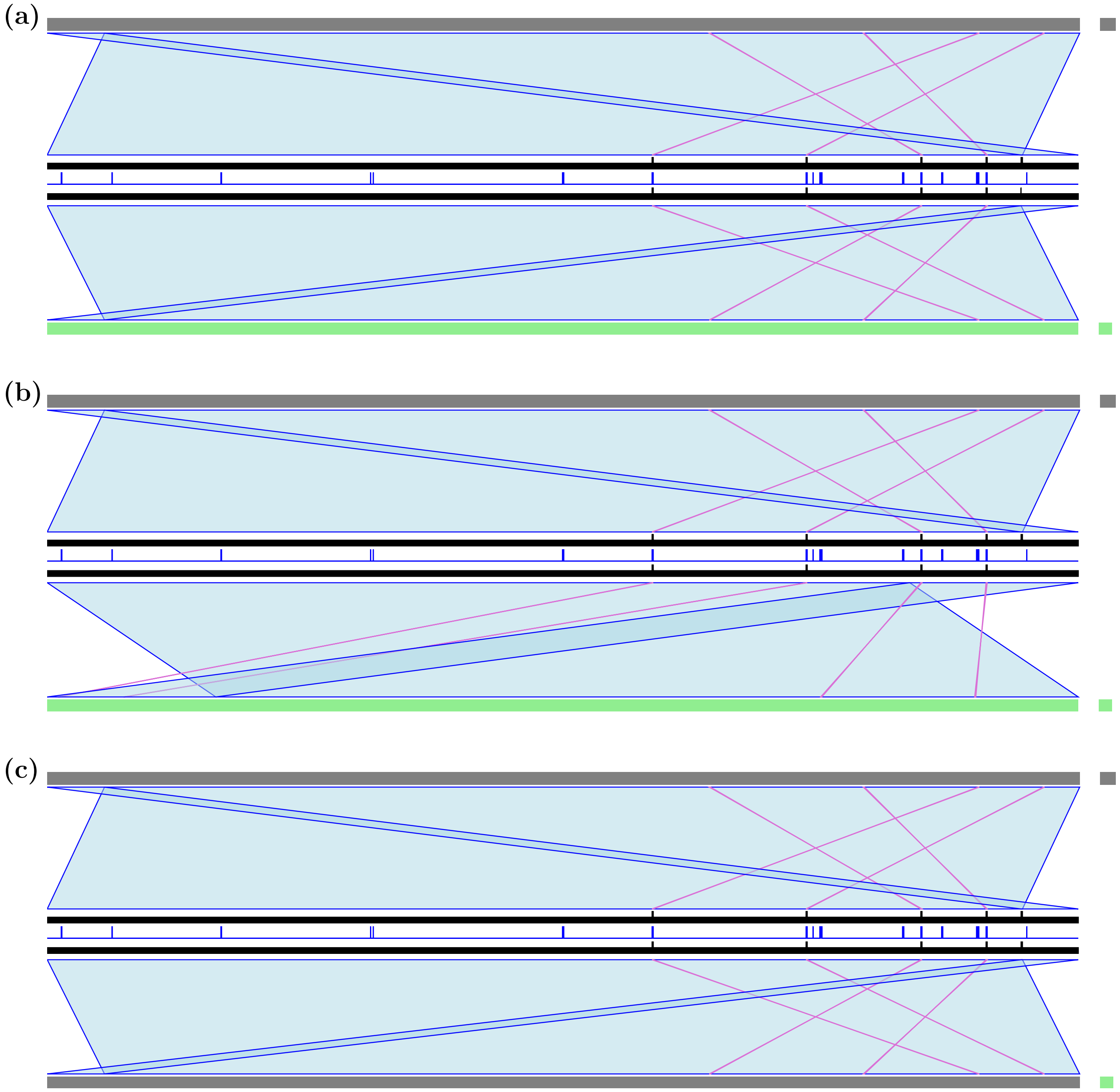

Figure S11: Comparison of circularizing NCTC sample NCTC13349 using (a) BLAST, (b) Circlator and (c) Minimus2.

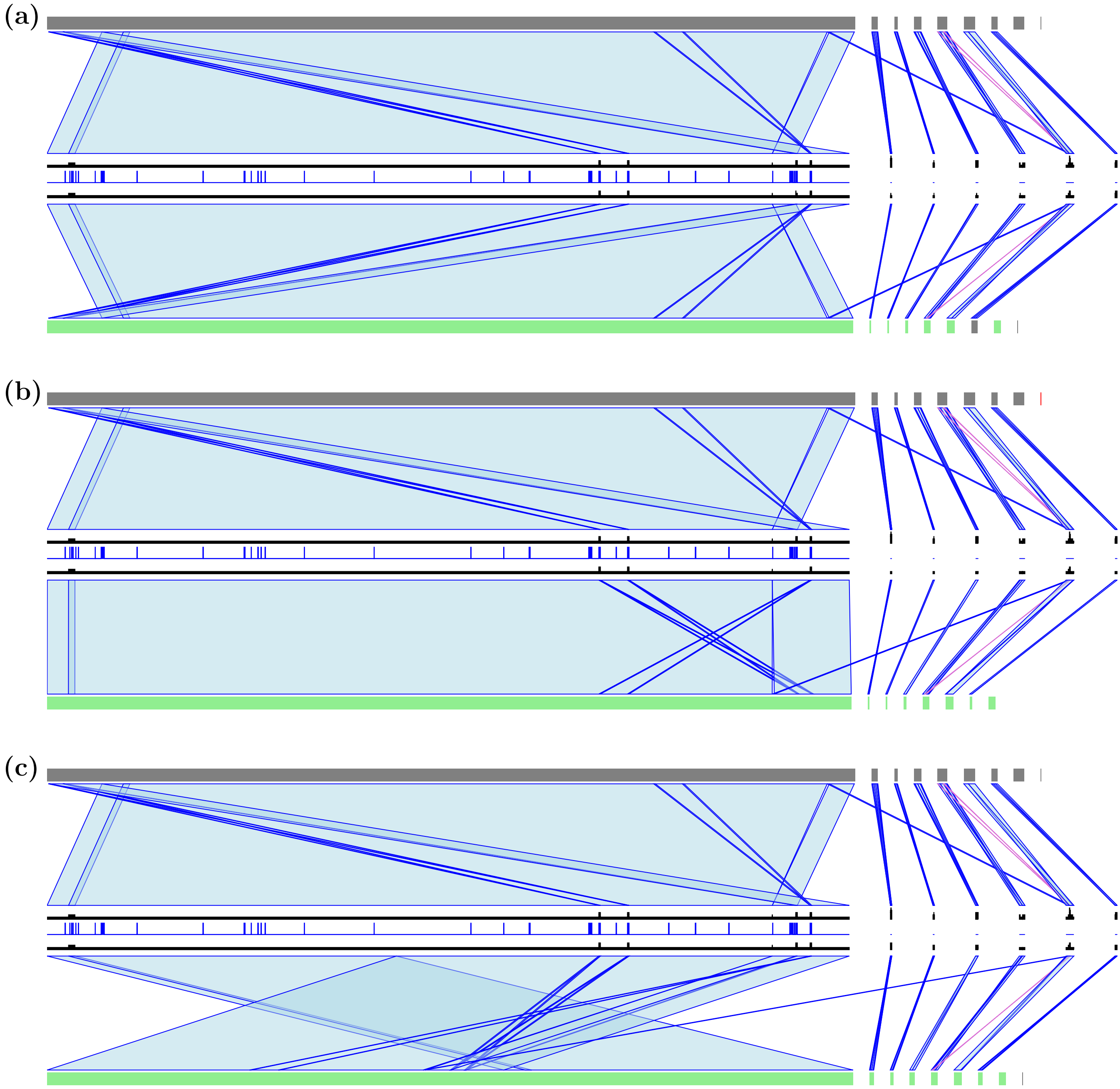

Figure S12: Comparison of circularizing NCTC sample NCTC13360 using (a) BLAST, (b) Circlator and (c) Minimus2.

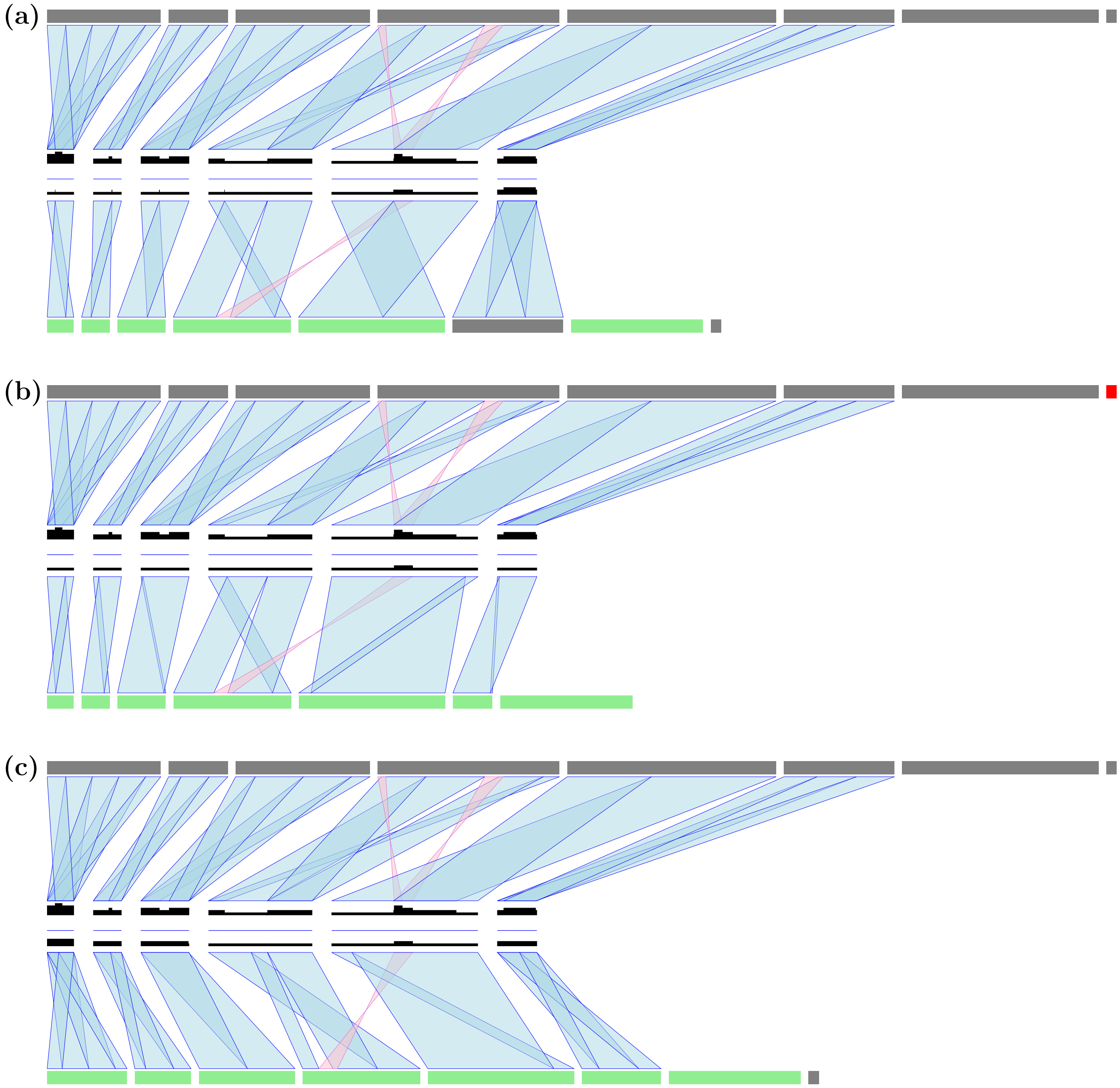

Figure S13: Comparison of circularizing the plasmids of NCTC sample NCTC13360 using (a) BLAST, (b) Circlator and (c) Minimus2.

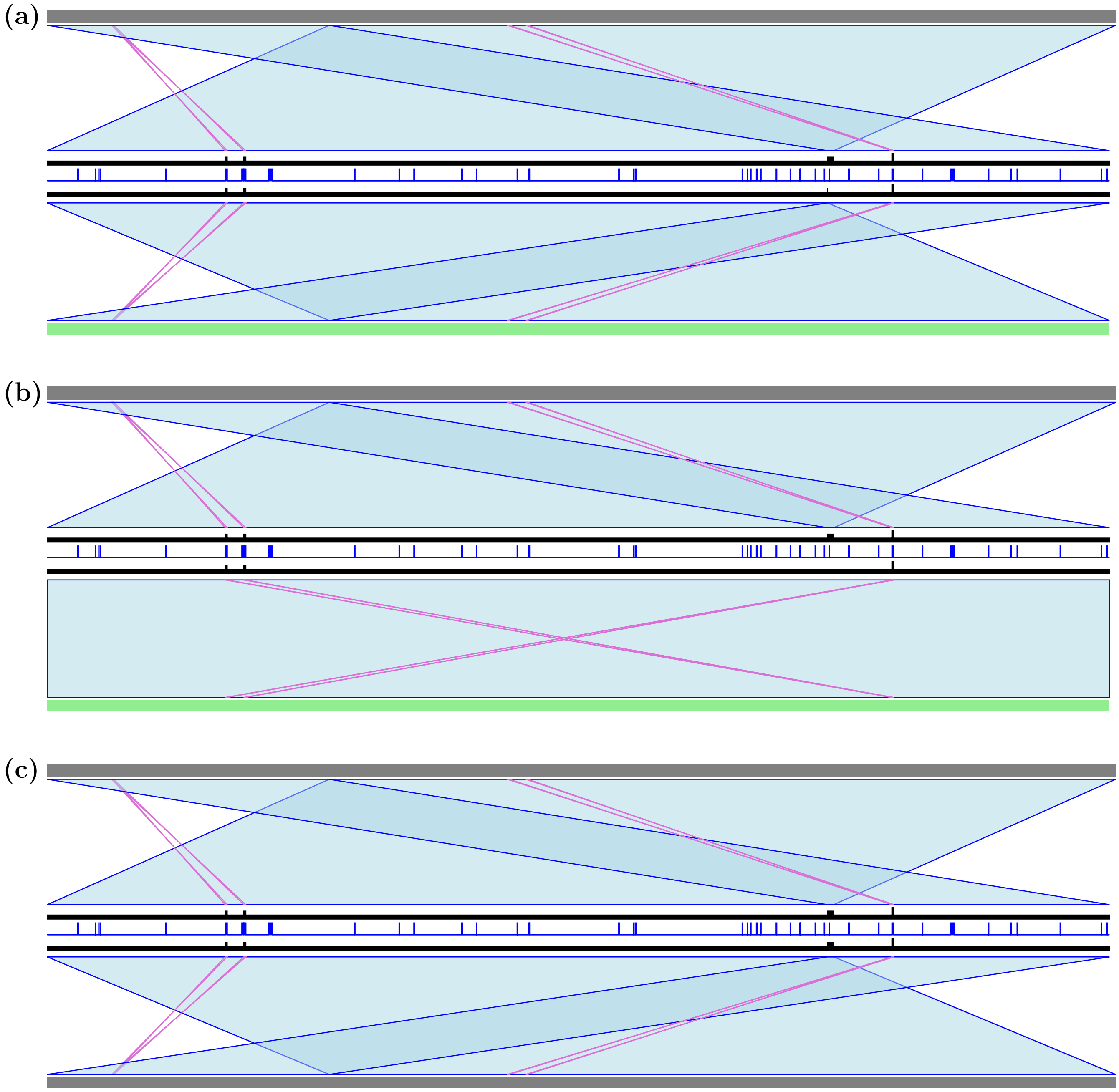

Figure S14: Comparison of circularizing NCTC sample NCTC13616 using (a) BLAST, (b) Circlator and (c) Minimus2.

(a)

(b)

(c)

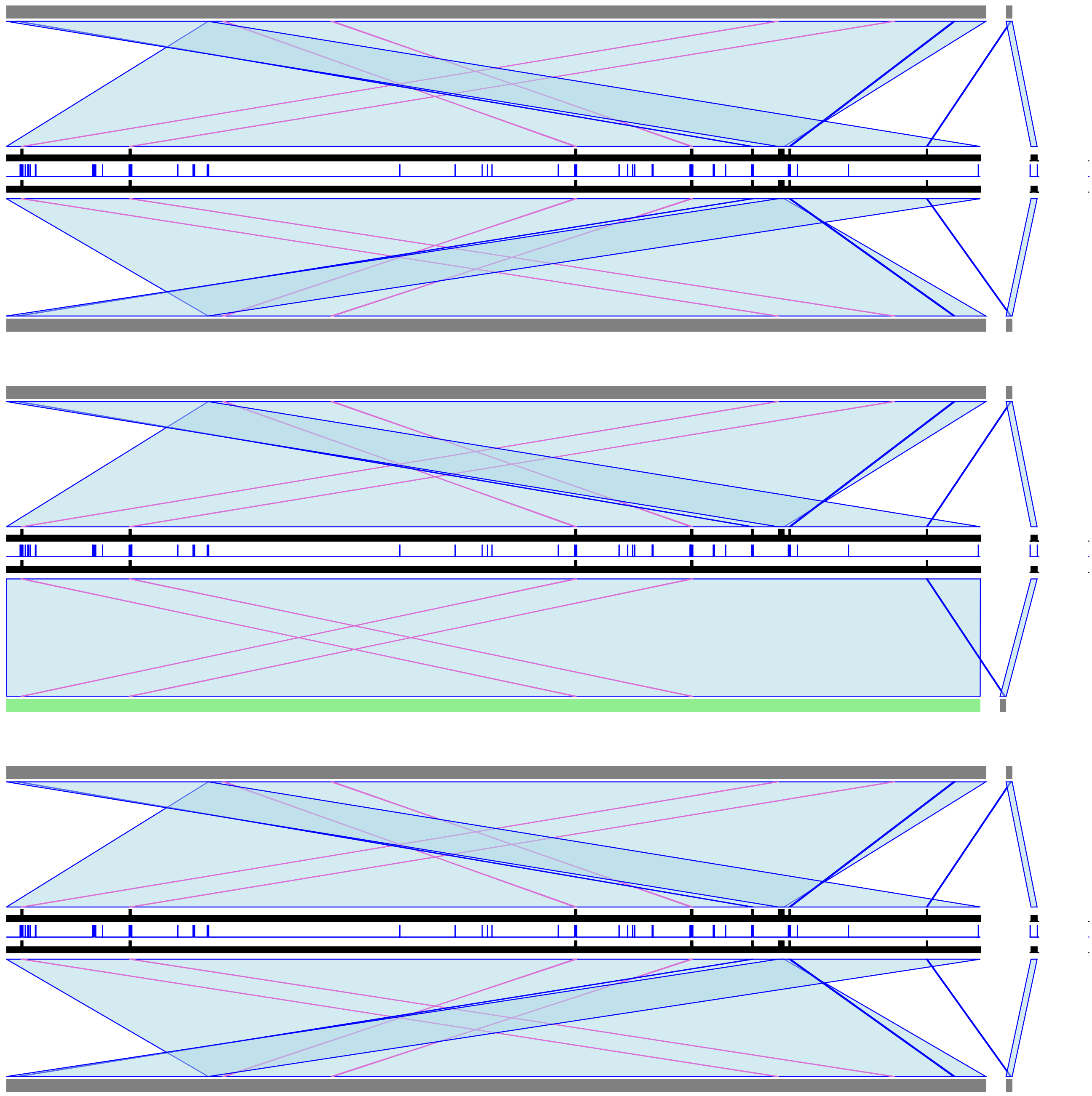

Figure S15: Comparison of circularizing NCTC sample NCTC13626 using (a) BLAST, (b) Circlator and (c) Minimus2.

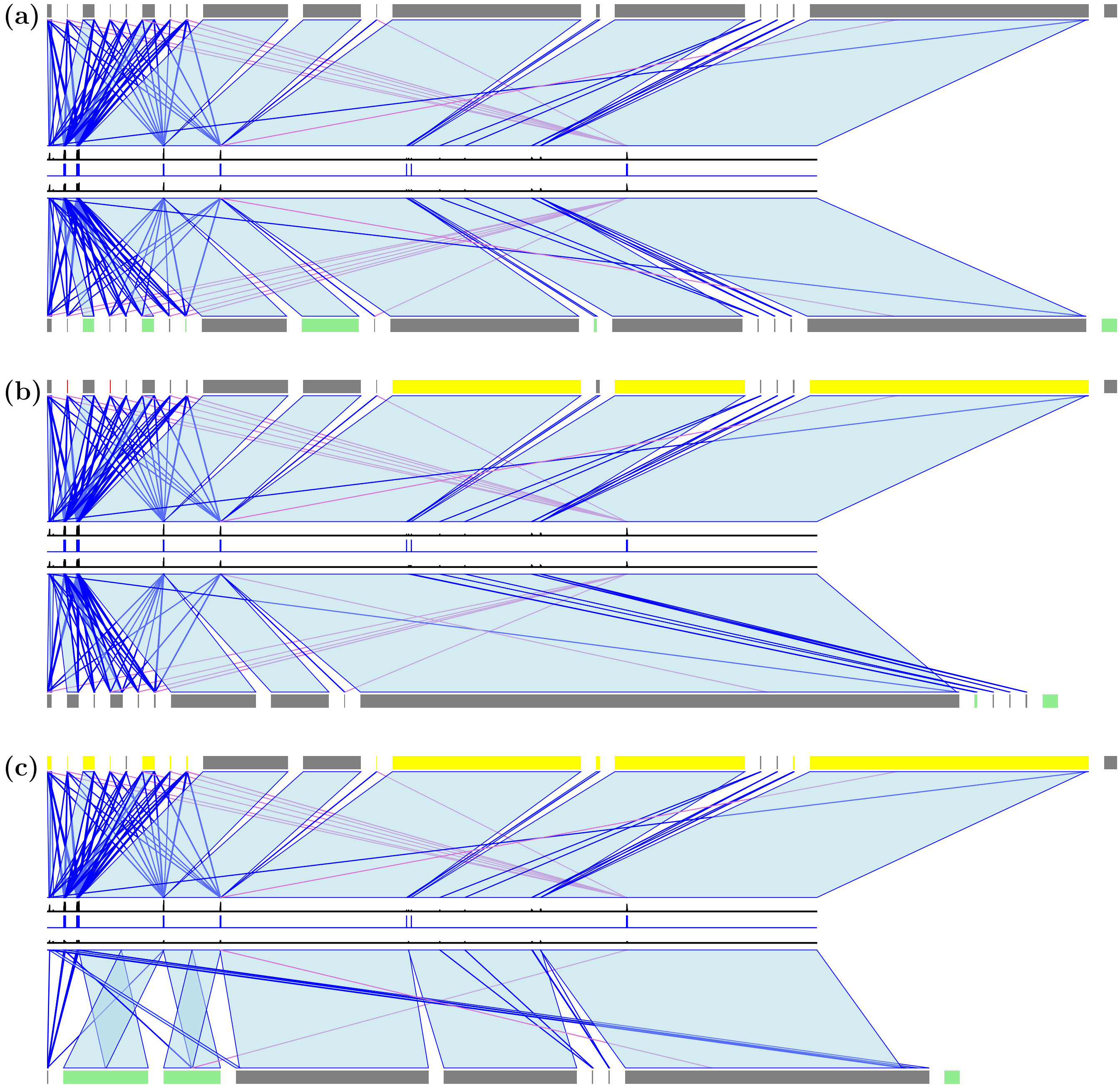

Figure S16: Comparison of circularizing NCTC sample NCTC3610 using (a) BLAST, (b) Circlator and (c) Minimus2.

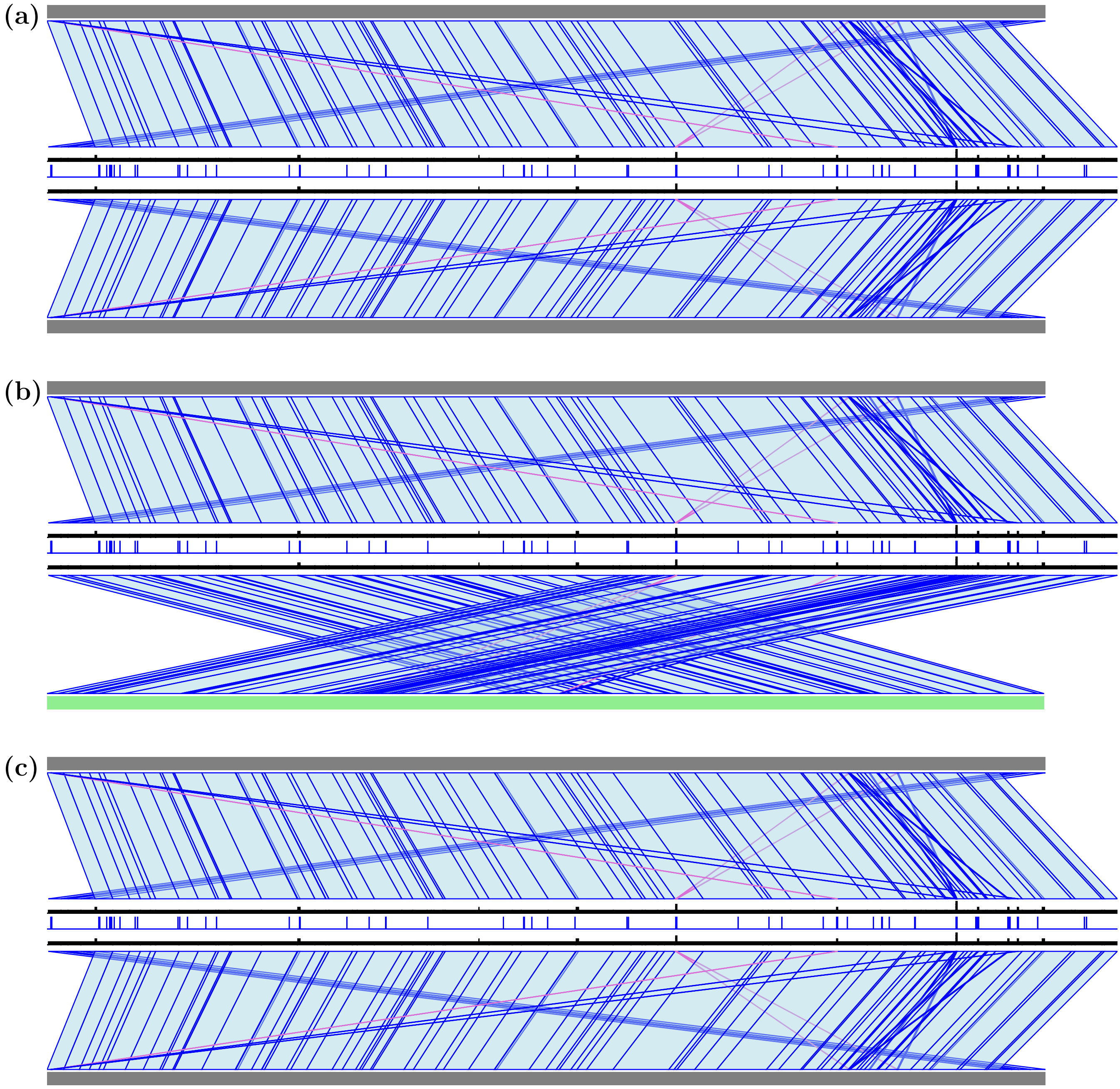

Figure S17: Comparison of circularizing the nanopore assembly using (a) BLAST, (b) Circlator and (c) Minimus2.

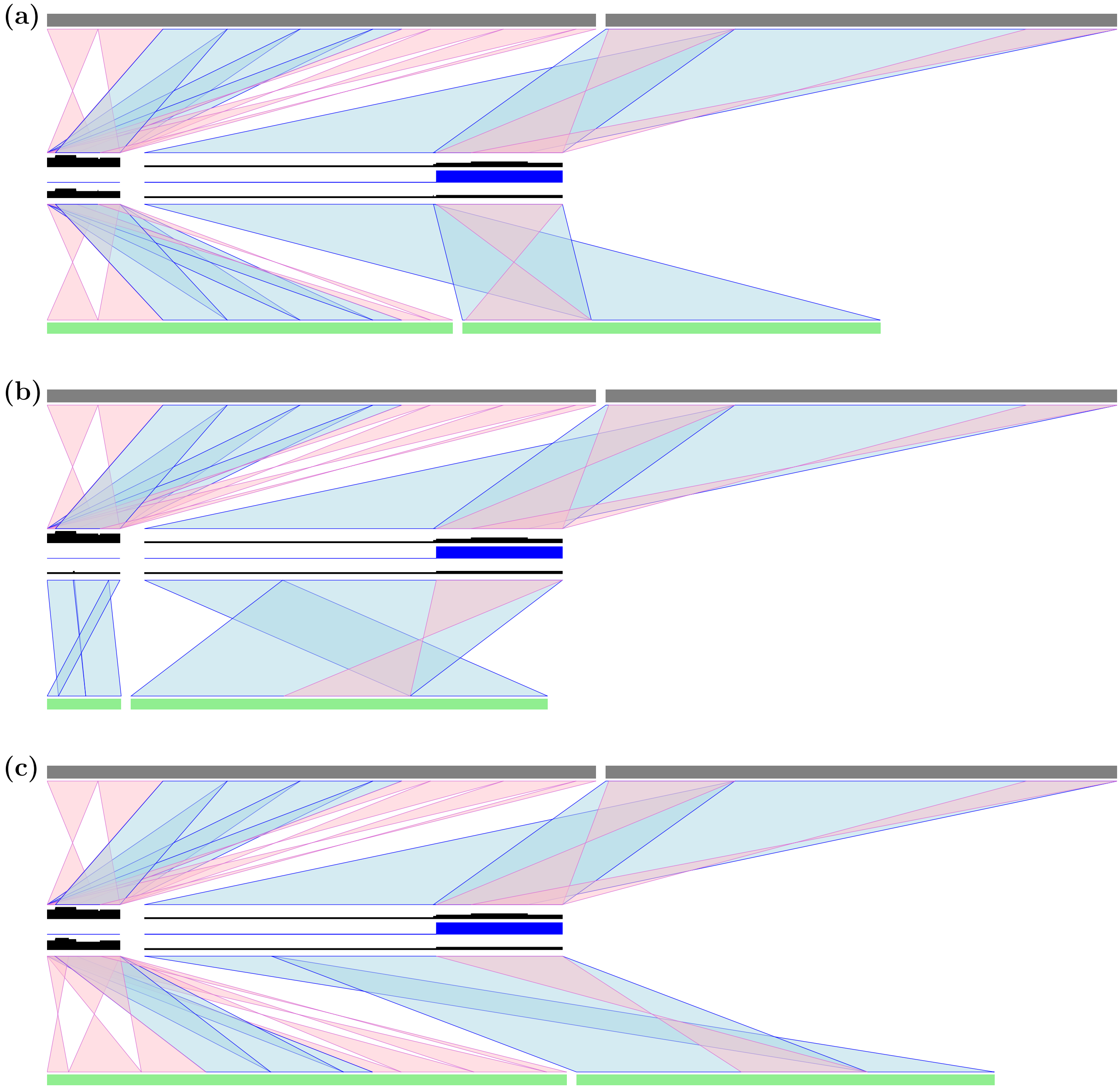

Figure S18: Comparison of circularizing the *P. falciparum* mitochondrion and apicoplast assemblies using (a) BLAST, (b) Circlator and (c) Minimus2.

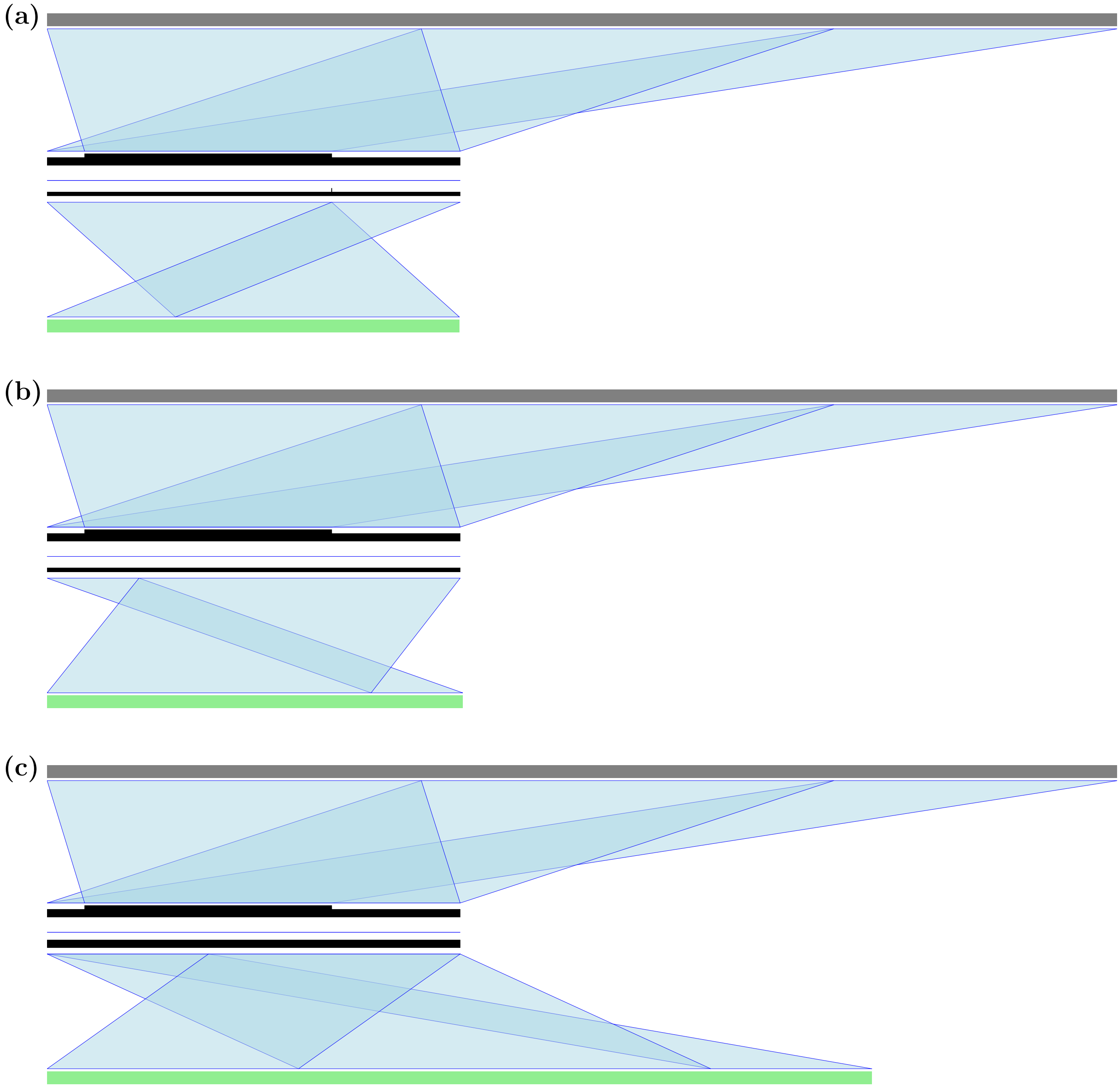

Figure S19: Comparison of circularizing the *H. sapiens* mitochondrion assembly using (a) BLAST, (b) Circlator and (c) Minimus2.

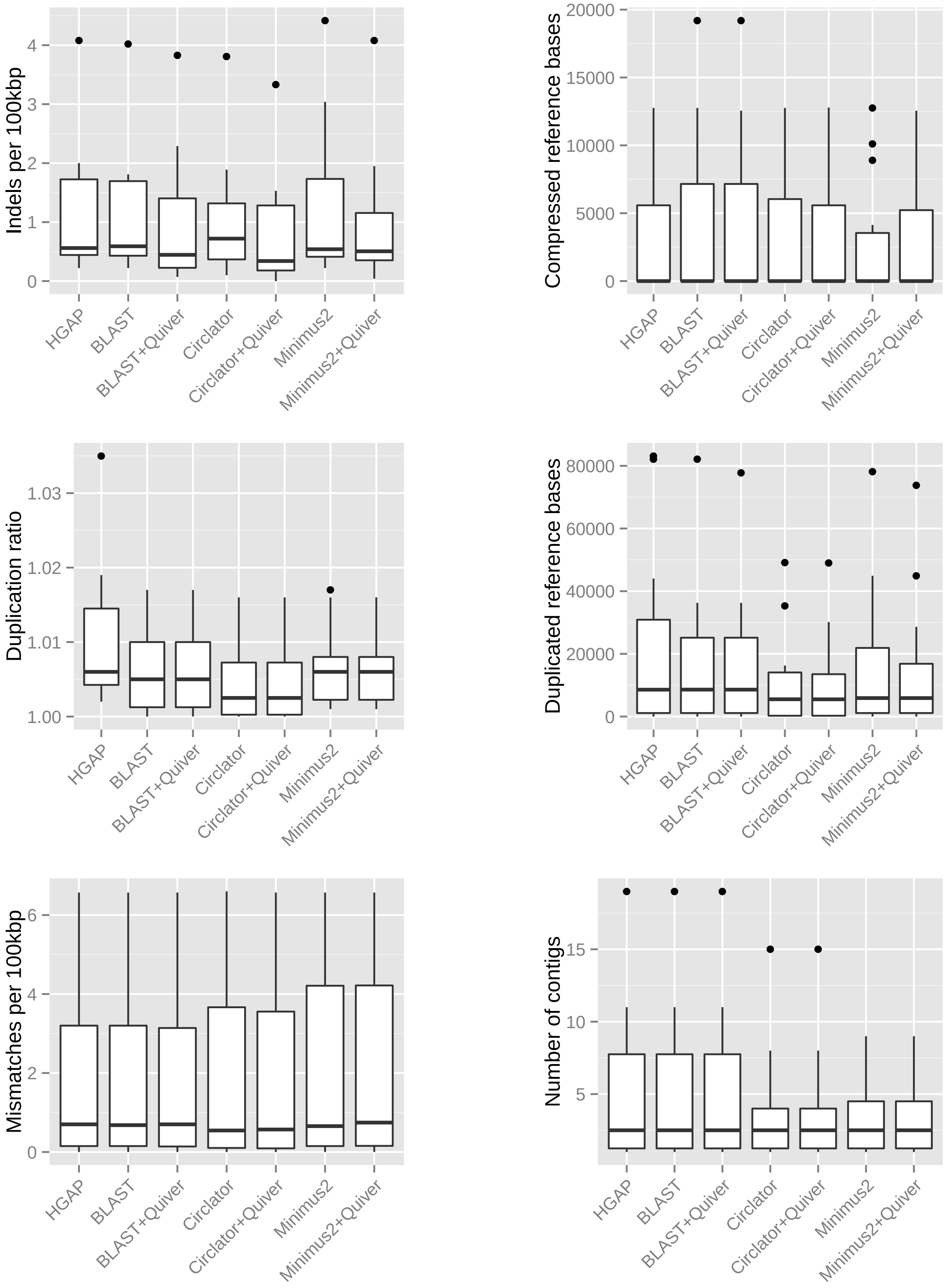

Figure S20: Summary of QUAST statistics on the 14 NCTC samples, comparing the input assemblies, the output of Circlator and the BLAST- and Minmus2-based methods, and the effect of Quiver on the output of those tools.

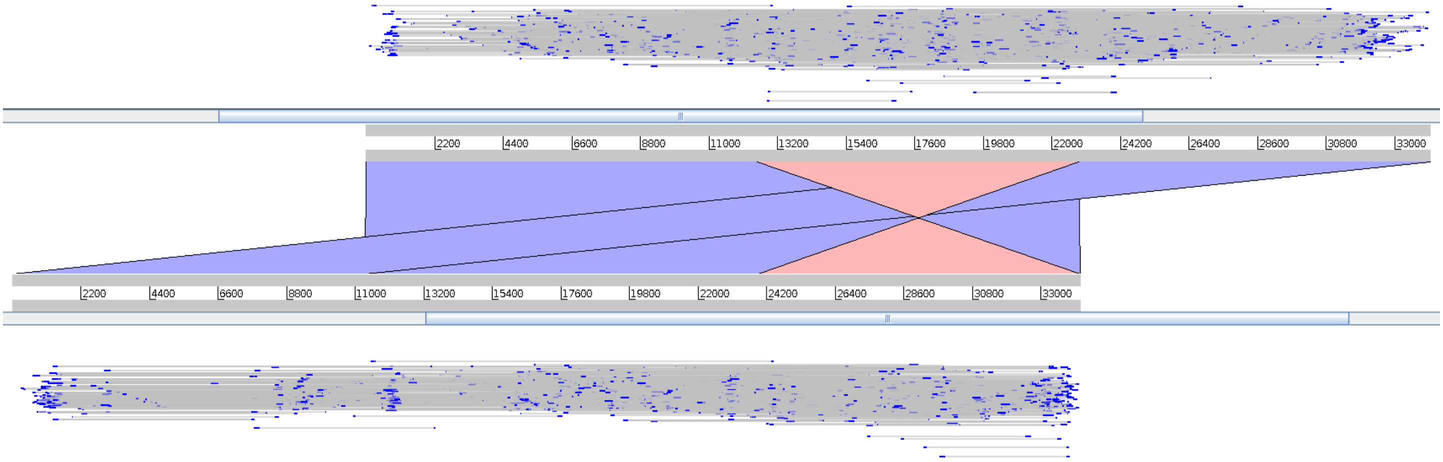

Figure S21: ACT screenshot showing a comparison of the reference (bottom) *P. falciparum* apicoplast genome and the output of Circlator (top). BLAST hits between the genomes are shown in blue (hits in the same direction) and red (in the opposite direction). Proper read pairs are shown in blue, with the two read within a pair joined by a grey line ('inferred size' view). The height of a read pair is determined by the distance between the reads (using a logarithmic scale).

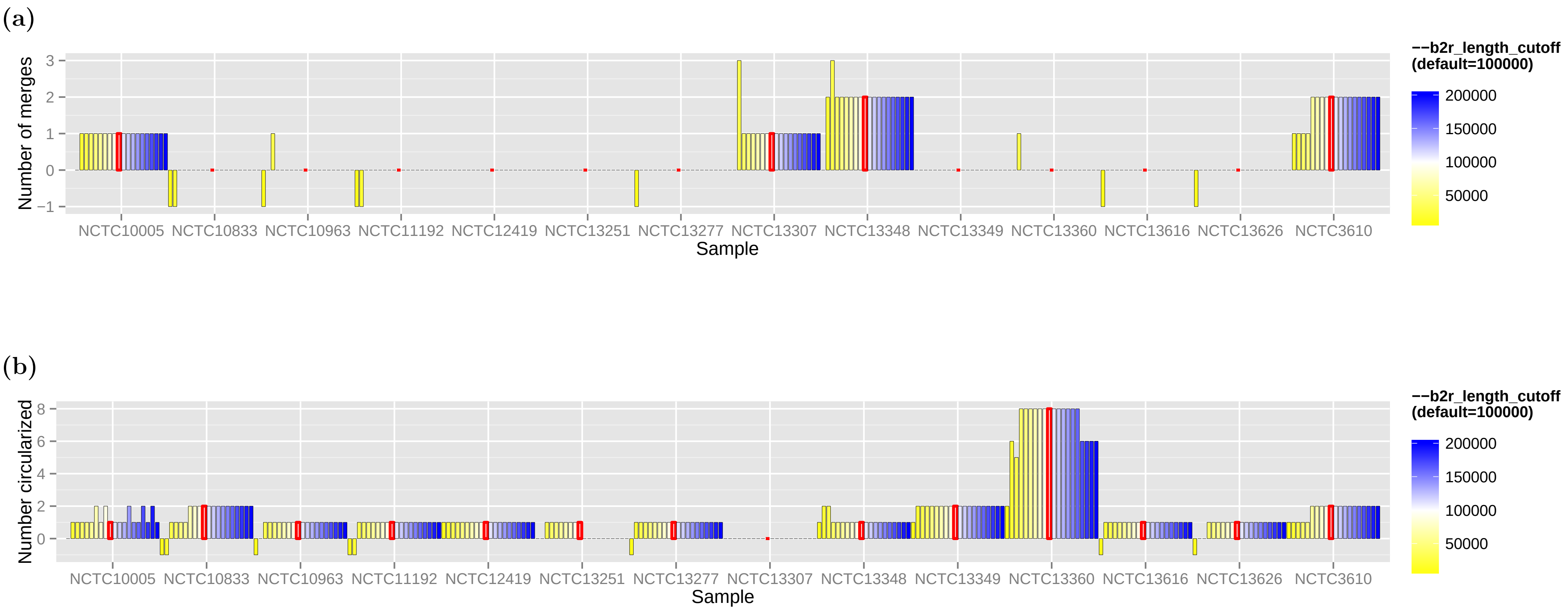

Figure S22: Results of varying the option `--b2r_length_cutoff`. (a) number of contig merges made before circularization. (b) number of contigs that were circularized. The bar corresponding to the default value is highlighted in red in each plot. A value of -1 means that the run did not complete due to the assembly failing.

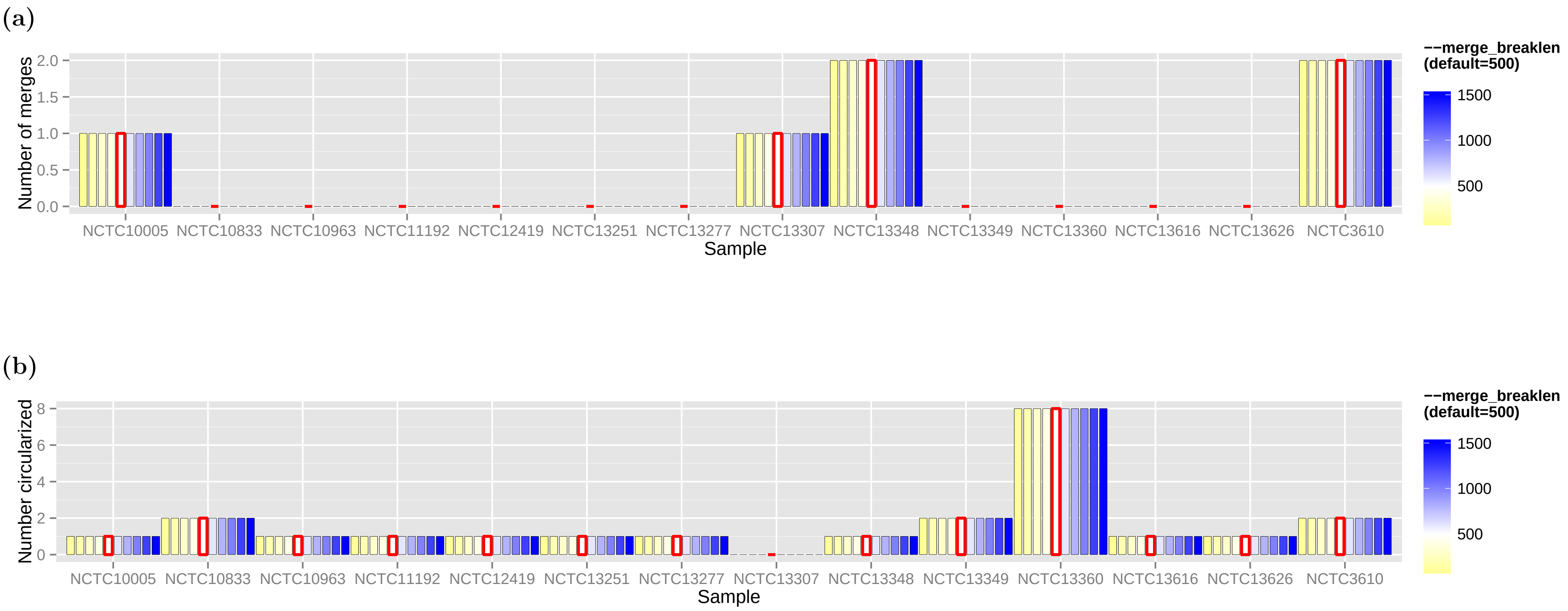

Figure S23: Results of varying the option `--merge_breaklen`. (a) number of contig merges made before circularization. (b) number of contigs that were circularized. The bar corresponding to the default value is highlighted in red in each plot.

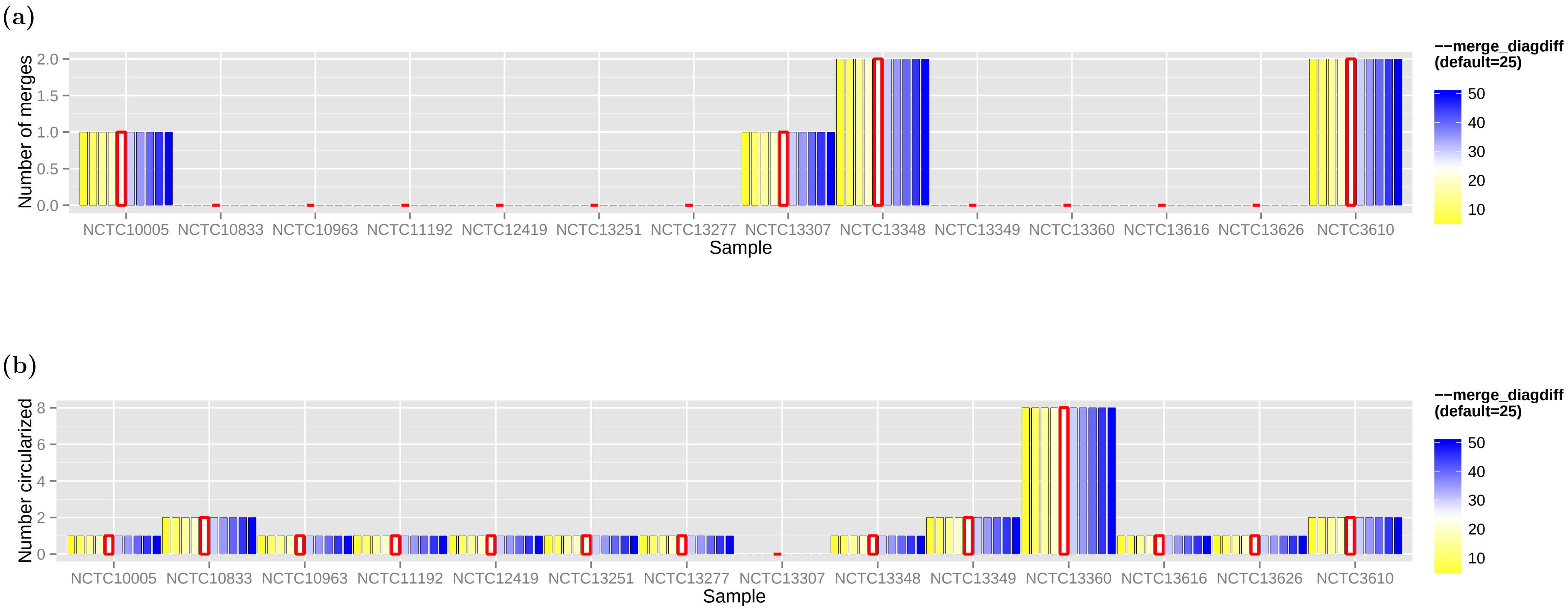

Figure S24: Results of varying the option `--merge_diagdiff`. (a) number of contig merges made before circularization. (b) number of contigs that were circularized. The bar corresponding to the default value is highlighted in red in each plot.

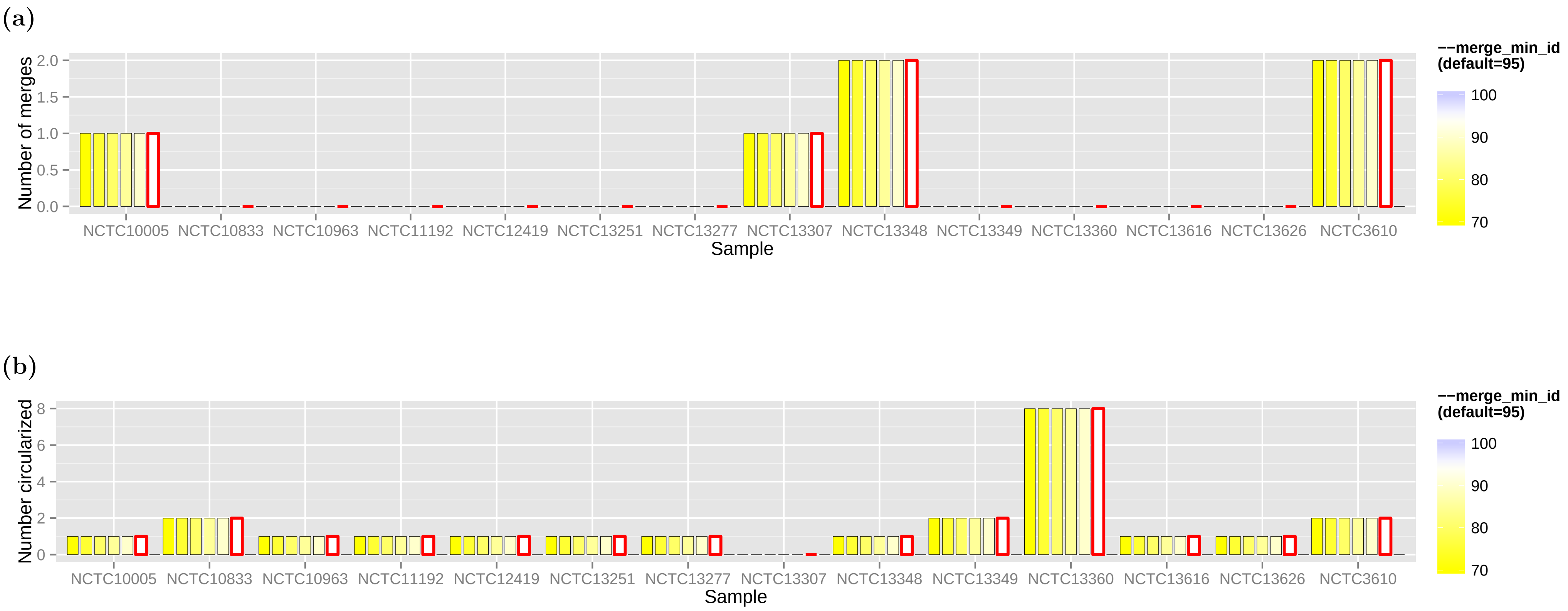

Figure S25: Results of varying the option `--merge_min_id`. (a) number of contig merges made before circularization. (b) number of contigs that were circularized. The bar corresponding to the default value is highlighted in red in each plot.

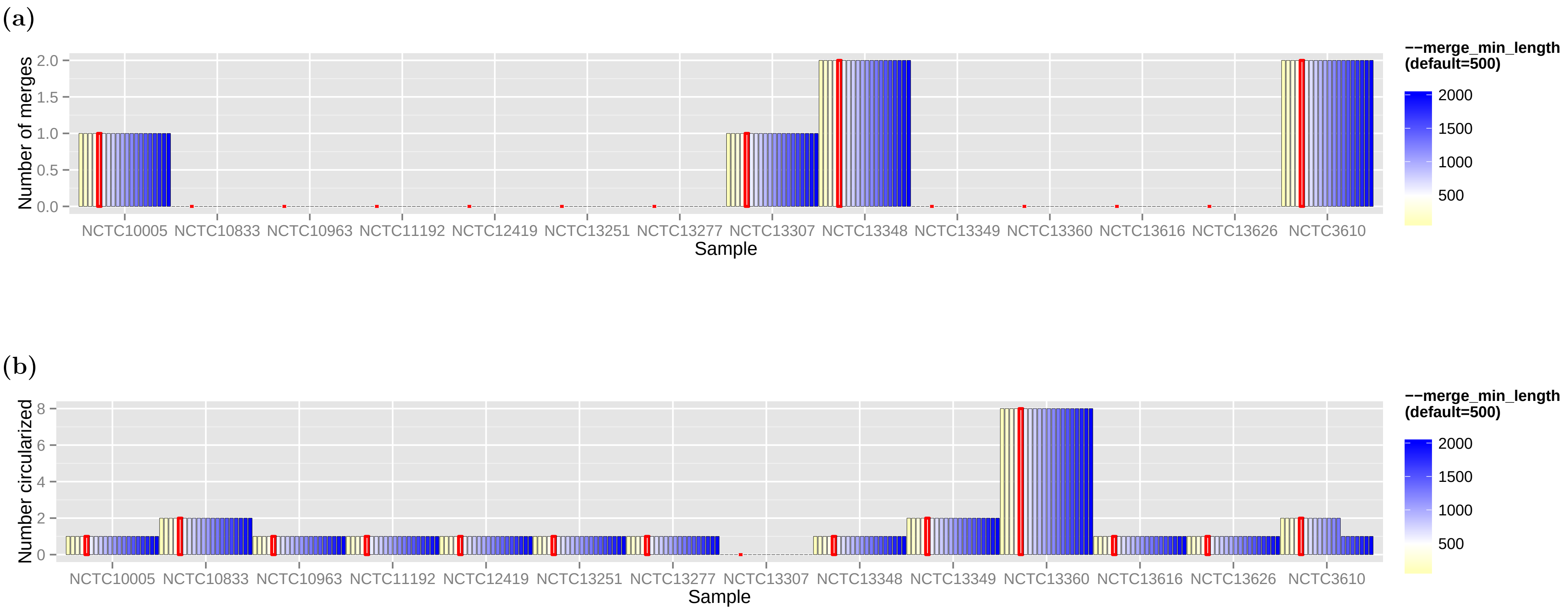

Figure S26: Results of varying the option `--merge_min_length`. (a) number of contig merges made before circularization. (b) number of contigs that were circularized. The bar corresponding to the default value is highlighted in red in each plot.

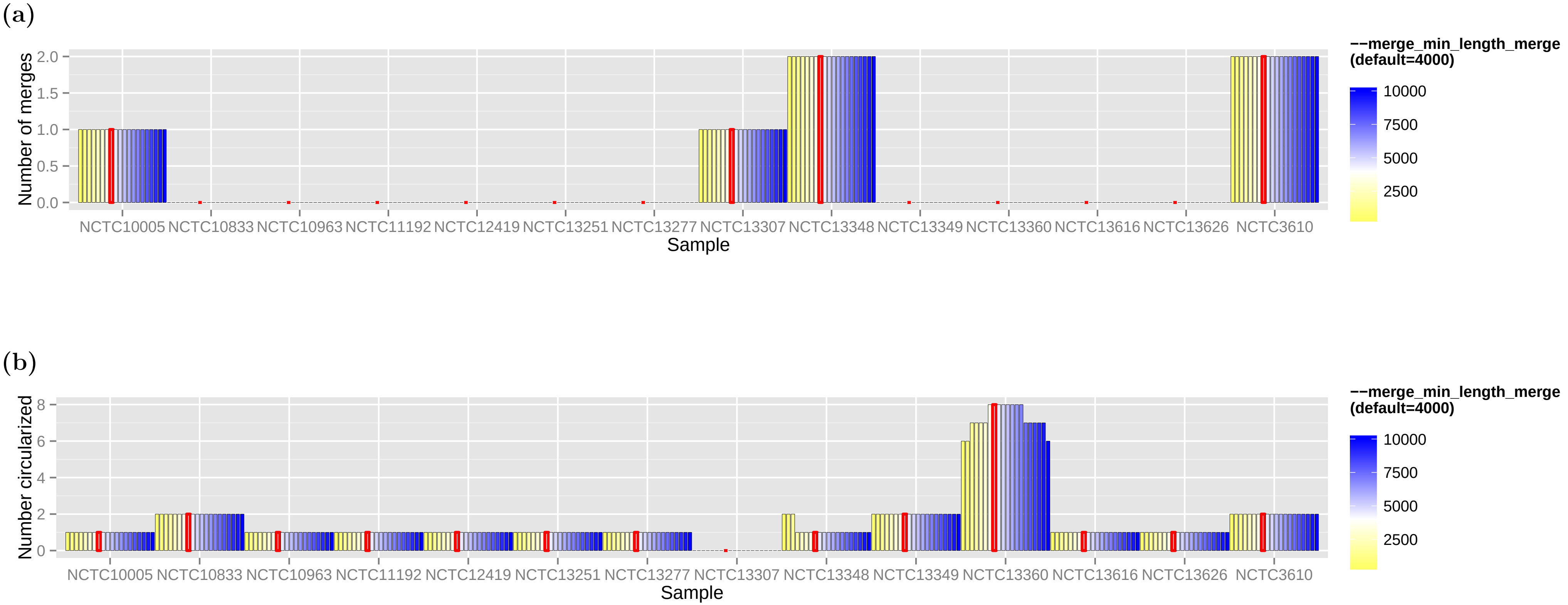

Figure S27: Results of varying the option `--merge_min_length_merge`. (a) number of contig merges made before circularization. (b) number of contigs that were circularized. The bar corresponding to the default value is highlighted in red in each plot.

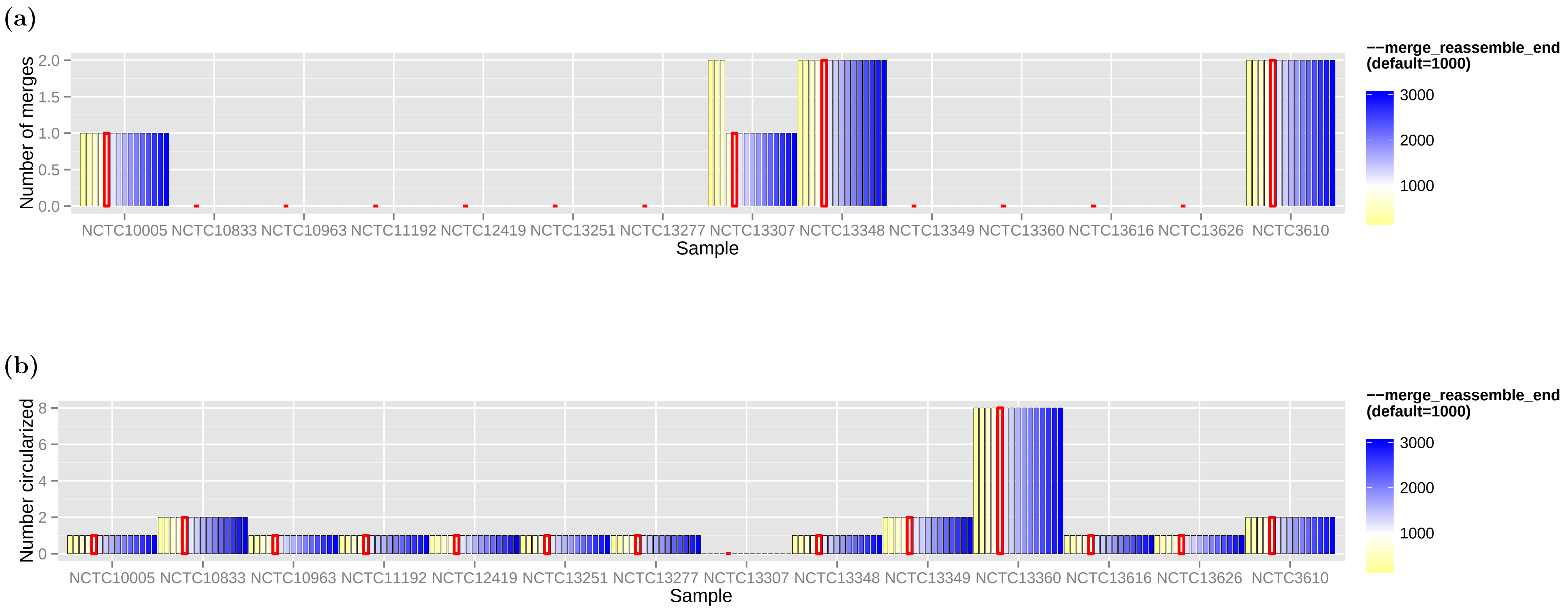

Figure S28: Results of varying the option `--merge_reassemble_end`. (a) number of contig merges made before circularization. (b) number of contigs that were circularized. The bar corresponding to the default value is highlighted in red in each plot.

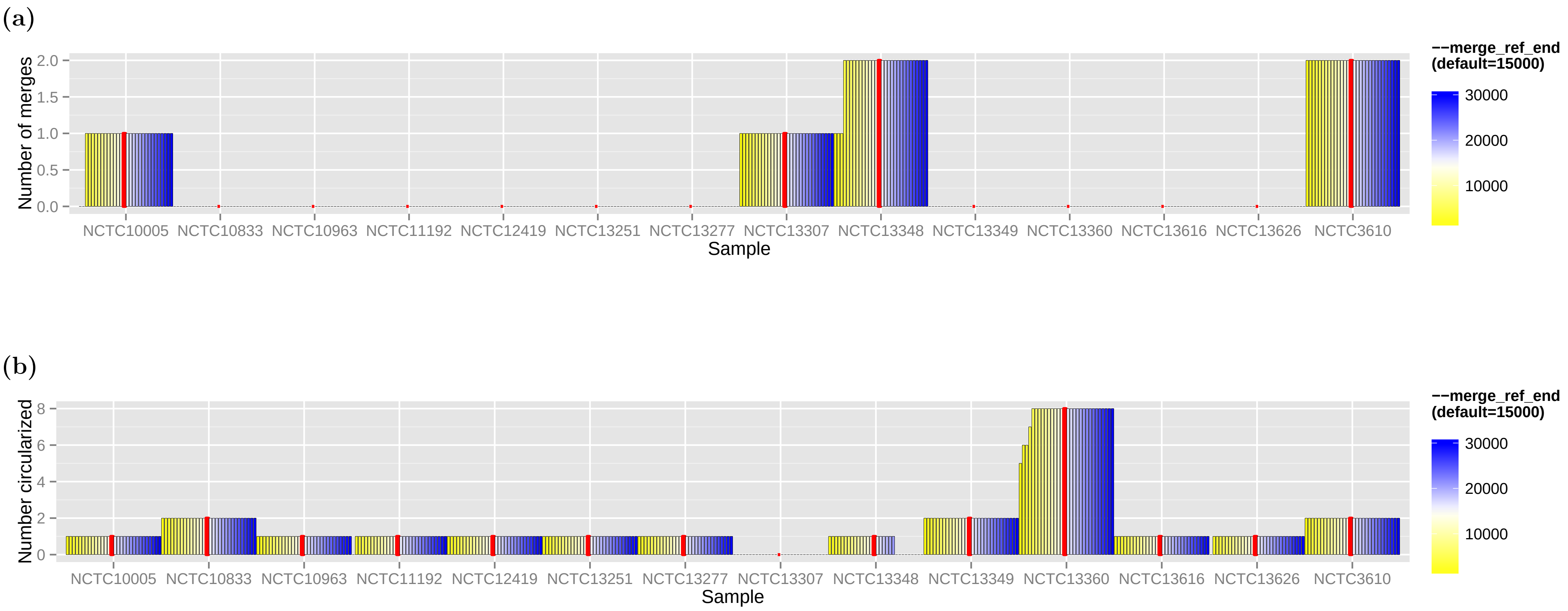

Figure S29: Results of varying the option `--merge_ref_end`. (a) number of contig merges made before circularization. (b) number of contigs that were circularized. The bar corresponding to the default value is highlighted in red in each plot.

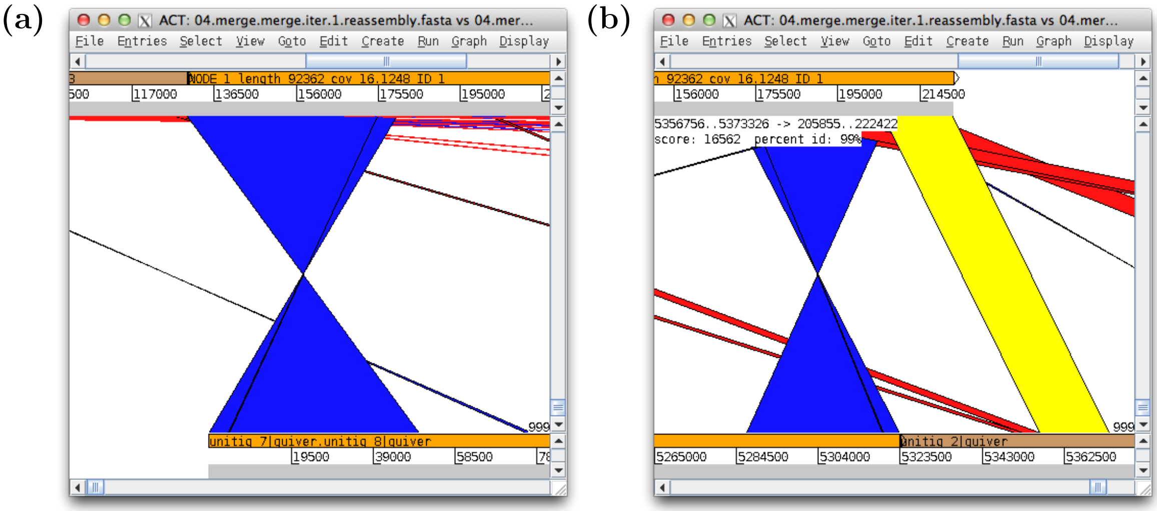

Figure S30: Example failed circularization for sample NCTC10005, when the parameter `--b2r_length_cutoff` was varied. In this case, the value was 100,000. Input assembly contigs are at the bottom, SPAdes reassembly contigs at the top, with `nucmer` matches shown between them in the middle. The start of the contig 'unitig_7', which should be circularized, is shown in (a) and its end in (b). The SPAdes contig 'NODE_1' contains a misassembly, so that instead of consisting of just the start and end of unitig_7, it also contains suprious sequence mathcing a different part of the genome (the match to this spurious sequence is shown in yellow).

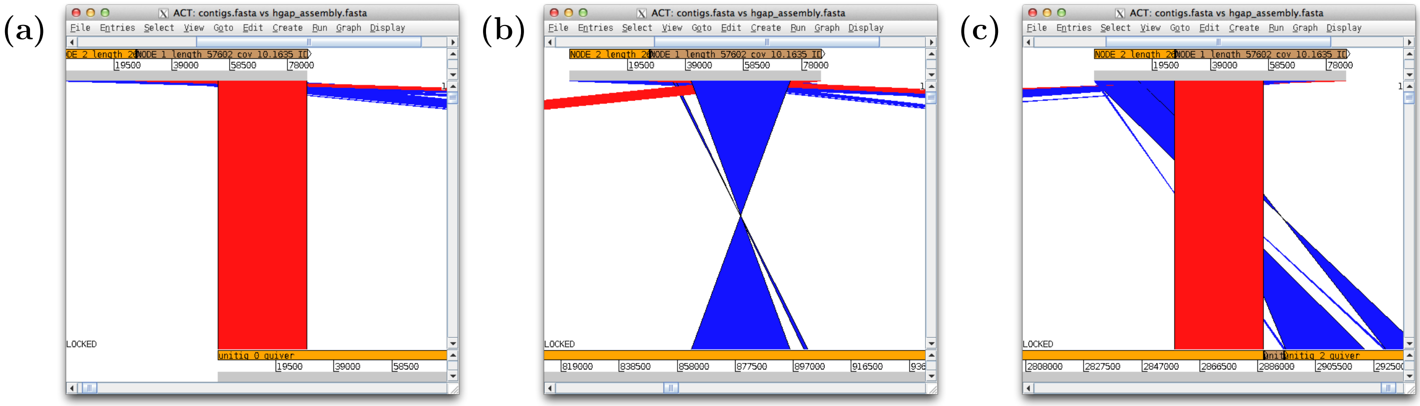

Figure S31: Example failed circularization for sample NCTC10833, when the parameter `--b2r_length_cutoff` was varied. In this case, the value was 60,000. Input assembly contigs are at the bottom, SPAdes reassembly contigs at the top, with `nucmer` matches shown between them in the middle. The start of the contig 'unitig_0', which should be circularized, is shown in (a), its middle in (b) and its end in (c). In this example, the SPAdes contig ('NODE_1', shown at the top of (a), (b) and (c) in dark brown) is not used to circularize unitig_0 because of the match shown in (b), which is longer than the matches to the ends of unitig_0.

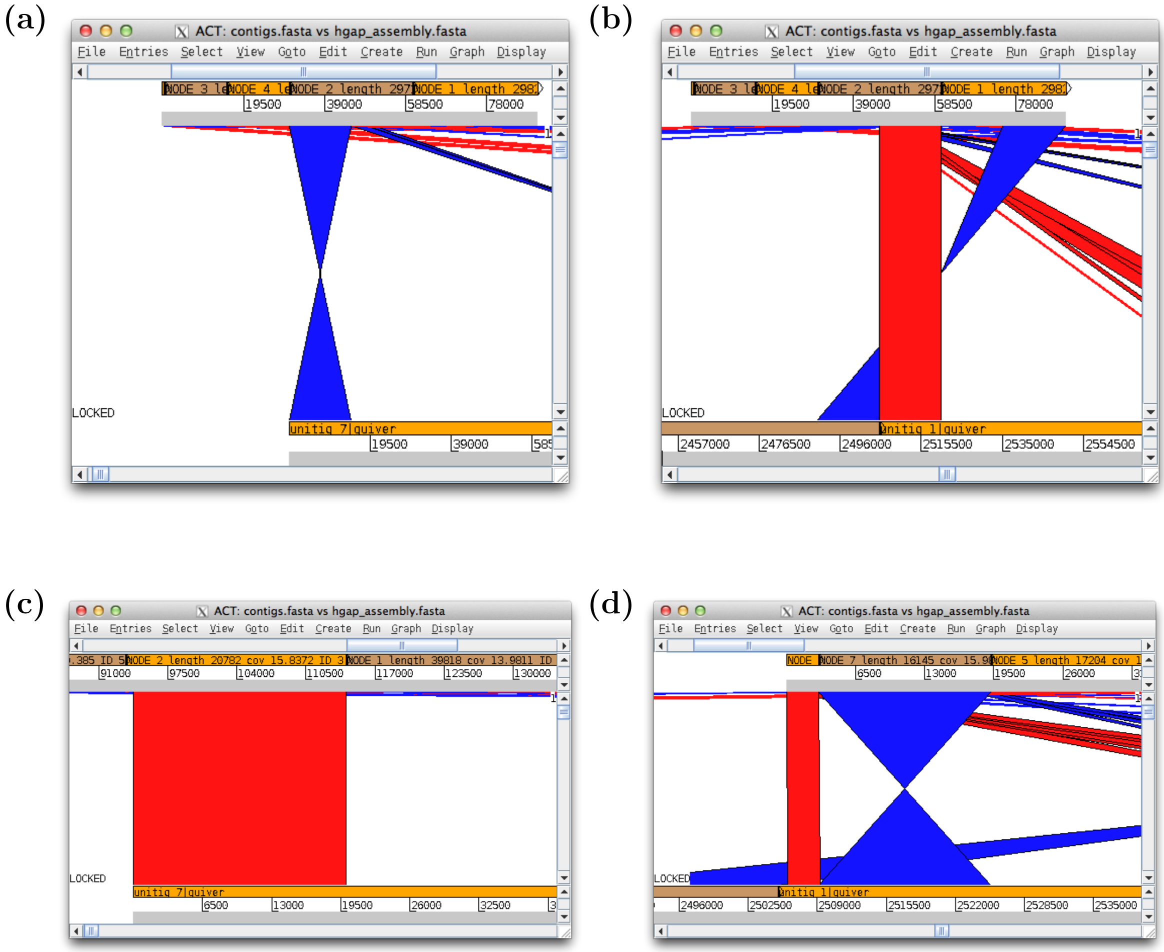

Figure S32: Example successful and failed merges for sample NCTC13307, when the parameter `--b2r_length_cutoff` was set to 30000 ((a) and (b)) and 40000 ((c) and (d)). The input HGAP contigs 'unitig_1' and 'unitig_7' (shown in the bottom of the screenshots) are merged with `--b2r_length_cutoff 30000` but not with `--b2r_length_cutoff 40000`. In (a) and (b) the SPAdes contig 'NODE_2' (in dark brown at the top) is can be used to merge 'unitig_1' and 'unitig_7'. In (c) and (d) SPAdes has assembled the ends of 'unitig_1' and 'unitig_7' into two separate contigs ('NODE_2' and 'NODE_7'), and therefore 'unitig_1' and 'unitig_7' cannot be merged by Circlator.

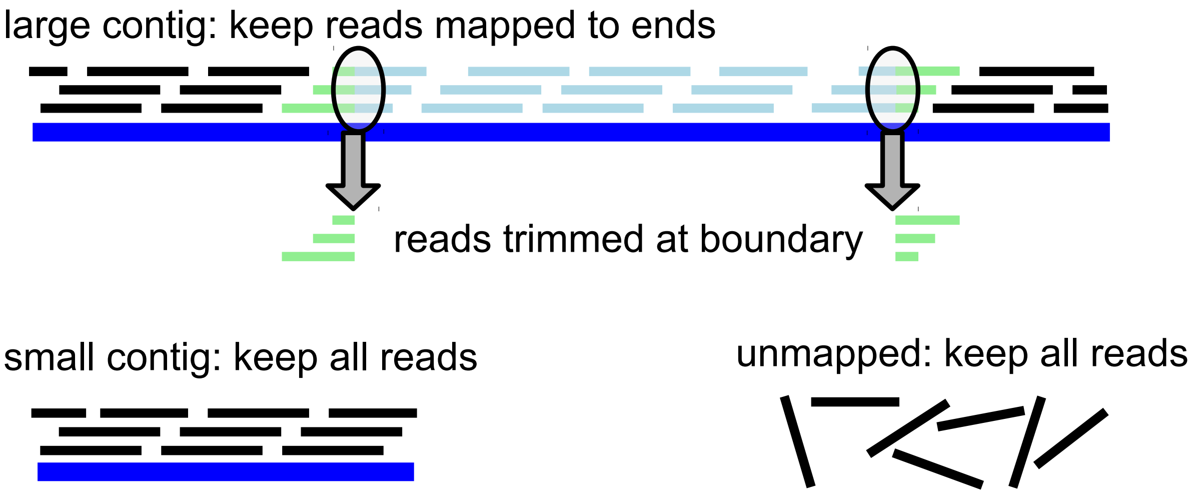

Figure S33: Read trimming and filtering used by Circlator for local assemblies

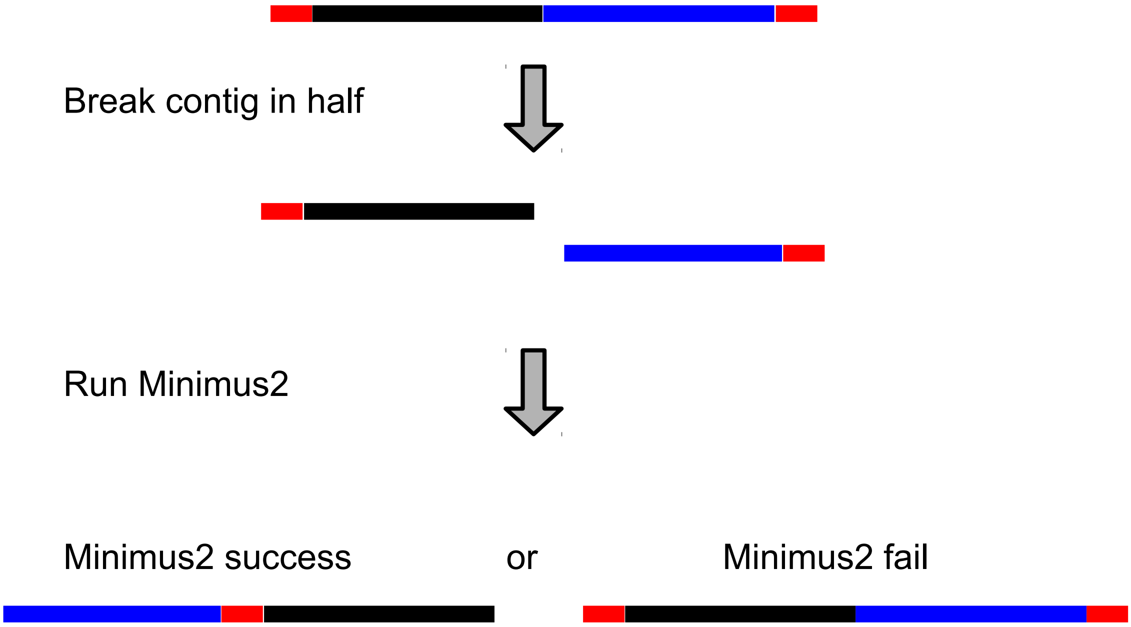

Figure S34: Using Minimus2 to circularize a single contig

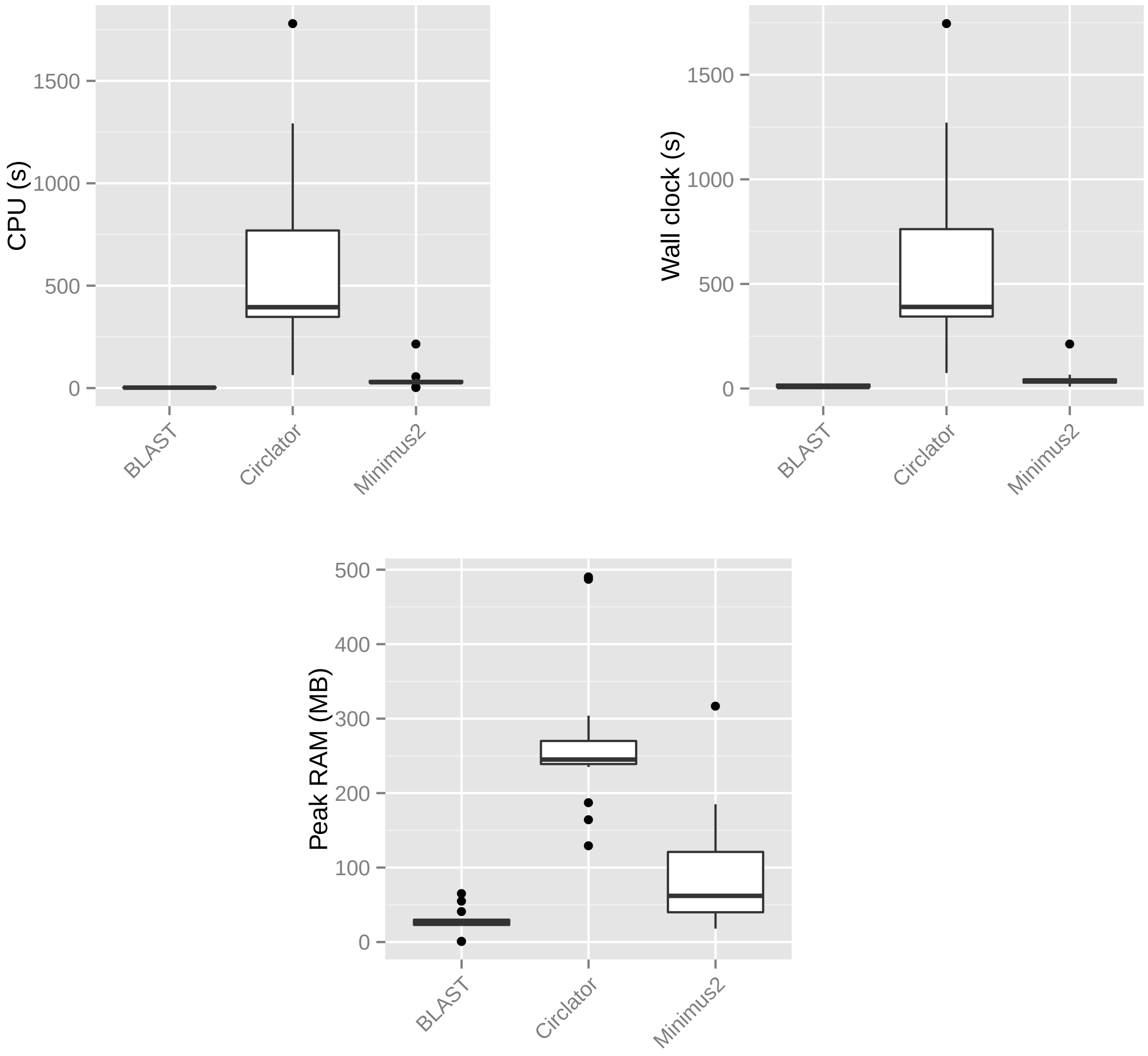

Figure S35: Summary of running time and RAM usage for all datasets
