# Supplementary material for: Circlator: automated circularization of genome assemblies using long sequencing reads
Source: Genome Biol. 2015 Dec 29;16:294. doi: 10.1186/s13059-015-0849-0 (PMC4699355; doi:10.1186/s13059-015-0849-0)
Supplement: Additional file 3 — A gzipped archive containing all scripts, corrected reads, assemblies, and other data necessary to reproduce the results. (gzipped archive 11GB) [file 13059_2015_849_MOESM3_ESM.pdf]

Additional file 3: A gzipped archive containing all scripts, corrected reads, assemblies and other data necessary to reproduce the results.
ftp://ftp.sanger.ac.uk/pub/pathogens/circlator/Supplementary_data/Circlator_supplementary_data.tar.gz
